# Human supplementation with *Pediococcus acidilactici* GR-1 decreases heavy metals levels through modifying the gut microbiota and metabolome

**DOI:** 10.1038/s41522-022-00326-8

**Published:** 2022-08-16

**Authors:** Pengya Feng, Jinfeng Yang, Shuai Zhao, Zhenmin Ling, Rong Han, Ying Wu, Ei-Sayed Salama, Apurva Kakade, Aman Khan, Weilin Jin, Weibing Zhang, Byong-Hun Jeon, Jingjing Fan, Minrui Liu, Tursunay Mamtimin, Pu Liu, Xiangkai Li

**Affiliations:** 1 Ministry of Education Key Laboratory of Cell Activities and Stress Adaptations, School of Life Science, Lanzhou University, Lanzhou, Gansu 730000 PR China; 2 College of Life Science and Technology, Gansu Agricultural University, Lanzhou, 730070 PR China; 3 Department of Occupational and Environmental Health, School of Public Health, Lanzhou University, Lanzhou, Gansu 730000 PR China; 4 Institute of Cancer Neuroscience, Medical Frontier Innovation Research Center, The First Hospital of Lanzhou University, The First Clinical Medical College of Lanzhou University, Lanzhou, 730000 PR China; 5 Department of Earth Resources and Environmental Engineering, Hanyang University, Seoul, 04763 South Korea

**Keywords:** Applied microbiology, Next-generation sequencing

## Abstract

Exposure to heavy metals (HMs) is a threat to human health. Although probiotics can detoxify HMs in animals, their effectiveness and mechanism of action in humans have not been studied well. Therefore, we conducted this randomized, double-blind, controlled trial on 152 occupational workers from the metal industry, an at-risk human population, to explore the effectiveness of probiotic yogurt in reducing HM levels. Participants were randomly assigned to two groups: one consumed probiotic yogurt containing the HM-resistant strain *Pediococcus acidilactici* GR-1 and the other consumed conventional yogurt for 12 weeks. Analysis of metal contents in the blood revealed that the consumption of probiotic yogurt resulted in a higher and faster decrease in copper (34.45%) and nickel (38.34%) levels in the blood than the consumption of conventional yogurt (16.41% and 27.57%, respectively). Metagenomic and metabolomic studies identified a close correlation between gut microbiota (GM) and host metabolism. Significantly enriched members of *Blautia* and *Bifidobacterium* correlated positively with the antioxidant capacities of GM and host. Further murine experiments confirmed the essential role of GM and protective effect of GR-1 on the antioxidative role of the intestine against copper. Thus, the use of probiotic yogurt may be an effective and affordable approach for combating toxic metal exposure through the protection of indigenous GM in humans.

ClinicalTrials.gov identifier: ChiCTR2100053222

## Introduction

Heavy metal (HM) contamination is a major environmental concern that poses considerable health risks to humans^1^. More than 20 million hm^2^ of cultivated land has been polluted by HMs worldwide^2^. Furthermore, millions of workers in China, Pakistan, and Europe are exposed to toxic metals in their work environment^3–5^. Approximately 1.8 million people work in brick kiln factories in Pakistan, where substantial levels of HMs such as cadmium (Cd), zinc (Zn), chromium (Cr), and nickel (Ni) are released^4^. The chemical industry accounts for 1.1% of the gross domestic production, with 115,700 employees being exposed to HMs in the European Union^5^. The consumption of HM-contaminated food is another major route for toxic metal accumulation in the body^6^. Excessive levels of HMs in the body induce oxidative stress (OS), resulting in various tissue lesions and elevated risks of cancer and other diseases in the long term^7^. Therefore, in addition to occupational exposure, dietary exposure to HMs may also be a long-term hidden threat to human health, affecting several people^2^. An effective treatment for HM toxicity is still lacking. Although chelation therapy is used to treat acute metal intoxication, it is associated with undesirable side effects^8^. A few dietary supplements (e.g., essential metals, vitamins, and phytochemicals) can alleviate HM-induced OS and tissue damage, although modestly^9,10^. Therefore, there is an urgent need for safe and effective methods to protect humans from the risk of HM exposure.

Probiotics are defined as living microorganisms that confer a health benefit on the host when administered in adequate amounts^11^. They exert beneficial effects on the gastrointestinal tract and immune system and also have other benefits such as the prevention of diabetes^12^, osteoporosis, depression, and anxiety and also the reduction of blood pressure^13–16^. The most attractive benefit of probiotics is that they reduce the accumulation of hazardous substances such as toxic metals, antibiotics, and pesticides in the host^17^. Most studies on the use of probiotics to detoxify toxic metals have been conducted in small animal models such as rats^18^, fruit flies^19,20^, honeybees^21^, the Nile tilapia^22^, and zebrafish^23^, whereas very few studies have been conducted in humans^24,25^. The first human trial using the probiotic strain *Lactobacillus rhamnosus* as an intervention agent to prevent HM accumulation indicated the efficacy of probiotics in preventing increases in toxic metal levels in the blood of human adults rather than children^26^. Similarly, another human trial demonstrated that an 8-week-long consumption of *L*. *plantarum* CCFM8610 markedly reduced blood Cd levels of the people belonging to towns near mining districts^27^. Current understanding of the protective mechanisms of probiotics against these xenobiotics primarily includes surface adsorption by the cell wall of probiotics^19,28^, protection of intestinal barrier^29,30^, regulation of enterohepatic circulation^27^, and modulation of indigenous gut microbiota (GM) of the host^2,20,23^. However, the specific roles of probiotics and cohabiting GM in the bioremediation of HMs remain obscure.

GM comprises 100 trillion microorganisms that inhabit the digestive tract of animals and has been recognized as an essential “barrier” that limits HM uptake^31–33^. The protective effects of probiotics against toxic metals (e.g., Cr, aluminum, and Cd) also include the modulation of GM^34–36^, emphasizing the essential role of GM in the detoxification of HMs. OS is a common toxic manifestation of HMs. Excessive production of reactive oxygen species (ROS) impairs the normal function of GM and the gut barrier^37,38^. A metagenomic study on the GM of mice exposed to arsenic reported that the abundance of genes involved in OS response was significantly upregulated, suggesting elevated OS and impaired function of the GM^39^. The antioxidant capacity of probiotics is crucial in maintaining the redox homeostasis. For instance, *Pediococcus* and *Lactobacillus* attenuate pesticide-induced ROS production in wildlife and humans through glutathione peroxidase-like 2 and catalase (CAT)^21,30^, and *Lactobacillus* sp. inhibits D-galactose-induced OS and GM dysbiosis in mice through its antioxidant activity^40–42^. Furthermore, two probiotic strains, *L. plantarum* TW1-1 and *P. acidilactici* GR-1, isolated from yak dairy significantly alleviate chromate toxicity in mice^2,36^. Therefore, we hypothesized that probiotics can maintain the intestinal redox homeostasis through their antioxidant capacity mediated through the protection the structural stability and normal metabolism and enhancement of GM resistance under HM stress.

Jinchang city is an important industrial zone in Gansu province, China, for its metal mining and smelting of copper (Cu), Ni, and cobalt, with ~45,000 workers in the industry and ~230,000 community dwellers living near the area^3^. This previous study revealed elevated levels of Cu and Ni in the urine of the aforementioned workers^3^. In the present study, we sought to investigate the potential of a probiotic yogurt enriched with the HM-resistant strain *P*. *acidilactici* GR-1^2^ to reduce toxic metal levels in the residents of Jinchang. We further explored the dynamics of GM in the probiotic-mediated gut remediation process and their implications on host metabolism.

## Results

### Probiotic yogurt, but not conventional yogurt, significantly decreased HM levels and improved serum biochemical indices over the 12 weeks of trial

A total of 152 occupational workers and 30 adult students were enrolled in this clinical trial (Table 1). The average levels of Cu and Ni in the blood of the worker group (1260 ± 213.7 and 4.960 ± 3.323 μg/L, respectively) were significantly higher (*p* < 0.0001, two-tailed Mann–Whitney test) than those in the blood of the student group (627.6563 ± 106.7 and 1.1 ± 0.3020 μg/L, respectively) (Supplementary Fig. 1a, d). The Cu levels in the blood of workers were similar to those previously reported in Thai occupational workers (1137.2 ± 258.0 μg/L)^43^. The Ni levels observed in this study were consistent with those of workers engaged in the leather manufacturing (4.18 μg/L)^44^ and surgical (4.66 μg/L) industries in Pakistan^45^. These levels noted in the worker group in the present study were, respectively, 1.26 and 4.51 times the normal values (1000 and 1.1 μg/L for Cu and Ni, respectively) reported by the Canadian Health Measures Survey 2007–2013^46^ (Supplementary Table 1). The results of Spearman’s correlation analysis performed in this study revealed that occupational exposure to Cu was significantly associated with age (*p* = 0.0246) but not with work duration (*p* = 0.0786) (Supplementary Fig. 2a, b). However, the blood Ni levels showed no significant association with work duration (*p* = 0.0619) or age (*p* = 0.0799) (Supplementary Fig. 2c, d).

**Table 1:** Demographics and baseline characteristics of the participants.

Characteristics	Conventional yogurt (*n* = 76)	Probiotic yogurt (*n* = 76)	Students (*n* = 30)	*P* value (Conventional vs Probiotic yogurt)
Average age, (range), years	40 (22–56)	37 (20–53)	22.4 (22–28)	0.0691
20–30, *n* (%)	18 (24%)	23 (30%)	30 (100%)	
31–50, *n* (%)	50 (66%)	50 (66%)	0 (0%)	
>50, *n* (%)	8 (11%)	3 (4%)	0 (%)	
Average work duration, (range), year	18.6 (2-37)	15.4 (1–36)	0	0.0525
Male, *n* (%)	61 (80%)	54 (71%)	8 (27%)	
Female, *n* (%)	15 (20%)	22 (29%)	22 (73%)	
Average body weight, (range), kg	67.37 (43–102.3)	67.48 (43–106)	59.05 (47–75)	0.9529
Average BMI^a^, (range), kg·m^−2^	23.37 (15.91–36.46)	23.6 (10.31–31.84)	20.97 (18.86–26.89)	0.7027
<25, BMI	55	53	22	
25–29.9, BMI	21	23	8	

^a^Body mass index (BMI) is the weight in kilograms divided by the square of the height in meters. No significant differences were observed between groups conventional yogurt with probiotic yogurt in any of the baseline characteristics. The *p* value was calculated with two tailed Student’s *t* test. Source data are provided as a Source Data file.

Results similar to the HM levels detected in the blood were obtained for urine samples (Supplementary Table 1; *p*_Cu_ < 0.0001 and *p*_Ni_ < 0.0001, two-tailed Mann–Whitney test). Both Cu and Ni levels in the feces of occupational workers were markedly lower than those of the control group (Supplementary Fig. 1c, f; *p*_Cu_ = 0.0146 and *p*_Ni_ < 0.0001). In addition to the high HM levels noted in blood samples, significantly decreased antioxidant enzyme activity and considerably enhanced OS response (*p*_CAT_ = 0.0008; *p*_malonaldehyde [MDA]_ < 0.0001) were noted in the serum of occupational workers compared with that of students (Supplementary Fig. 1g, h). Moreover, increased levels of proinflammatory cytokines (Supplementary Fig. 1i, j; *p*_interleukin [IL]-1β_ < 0.0001 and *p*_IL-6_ < 0.0001) and decreased levels of anti-inflammatory cytokines were detected in the worker group (Supplementary Fig. 1k, l; *p*_IL-4_ = 0.0177 and *p*_IL-10_ = 0.0007). The results suggested that exposure to HMs in manufacturing industries poses health risks through increased OS and inflammation.

**Fig. 1:**
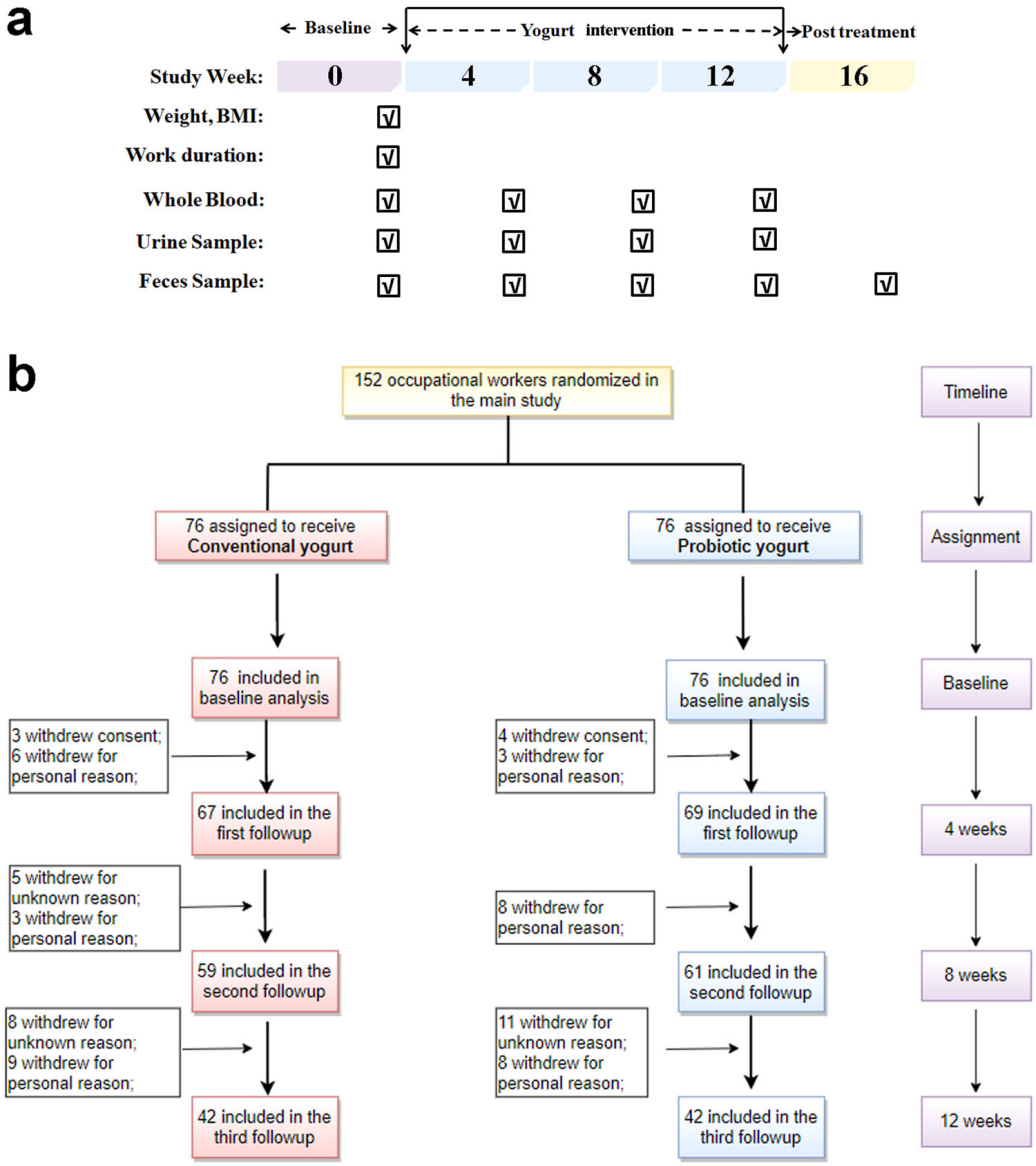
Flow diagram of the cohort of this trial. **a** Trial design. **b** Flow diagram of participant recruitment.

**Fig. 2:**
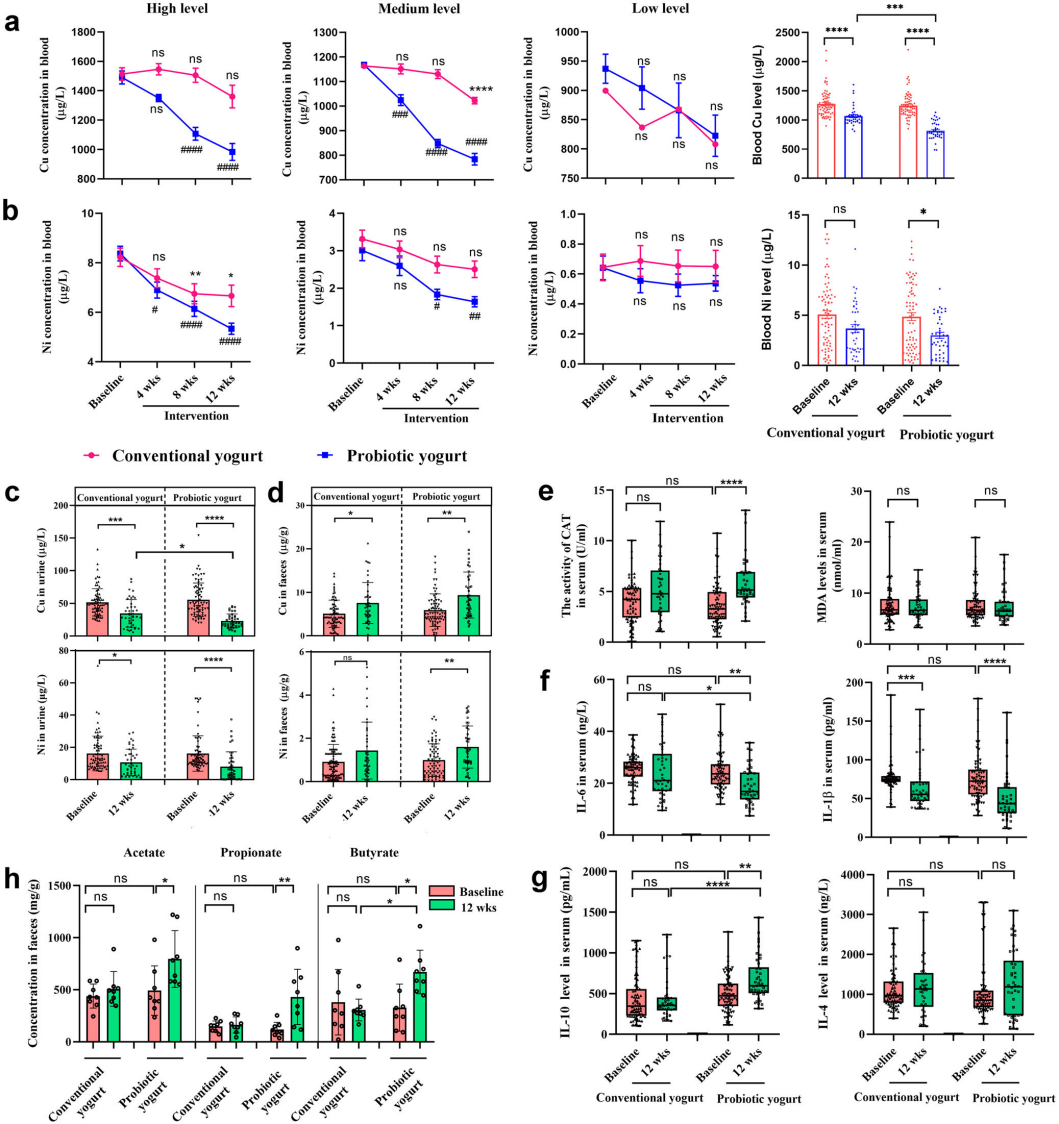
Probiotic yogurt significantly decreased heavy metal levels, altered blood biochemical indices, and increased SCFA production in the worker group. Blood Cu (**a**) and Ni (**b**) levels in the high-, medium-, and low-level subsets and total levels of participants in the two groups over the trial period. Changes in urine Cu and Ni levels (**c**) and fecal Cu and Ni (**d**) throughout the trial. Serum CAT activity and MDA level (**e**), IL-6 and IL-1β levels, and (**f**) IL-10 and IL-4 levels (**g**) of the participants. **h** Changes in fecal levels of acetate, propionate, and butyrate after yogurt supplementation. For **a**–**d**, **h**, data are expressed as means ± standard errors of the means. SCFAs were expressed as per gram wet feces. For **e**–**g**, box limits represent 25 and 75 percentiles; the line within the box indicates median; the whisker represents the minimum and maximum points. **a**–**g** baseline (*n* = 35) vs. 12 weeks (*n* = 42); **h** *n* = 8 participants in each group. Statistical analysis was performed using Kruskal–Wallis test with Dunn’s post hoc analysis in subgroups. ns, not significant; * (#), *p* < 0.05; ** (##), *p* < 0.01; *** (###), *p* < 0.001; ****(####), *p* < 0.0001. **a**, **b** * represents significance in the conventional yogurt group; # represents significance in the probiotic yogurt group at the same time point. CAT catalase, MDA malonaldehyde, IL interleukin. Source data are provided as a source data file.

The 152 worker participants of this study were randomly categorized into conventional and probiotic yogurt groups (Fig. 1 and Supplementary Table 2). Baseline characteristics of the participants did not vary significantly between the two groups; the characteristics assessed included work duration (Table 1; *p* = 0.0525), blood Cu and Ni levels, and other hematological parameters (Supplementary Table 3; *p*_Cu_ = 0.4171 and *p*_Ni_ = 0.6982). Considering the heterogeneity in the baseline blood Cu and Ni levels of the two groups, we stratified each group into three sets—low, medium, and high levels of HMs in the blood—based on a reference value^46^ (Supplementary Table 4). In the high-level set, participants who consumed probiotic yogurt showed significantly decreased blood Cu levels (1490 ± 44.56 vs. 1107 ± 43.09 μg/L; *p* < 0.0001, Kruskal–Wallis test with Dunn’s post hoc analysis) after 8 weeks of intervention compared with the baseline levels; on the other hand, participants who consumed conventional yogurt exhibited only a slight decrease in these levels during the same period (*p* > 0.9999) (Fig. 2a). In the medium-level set, a significant decrease was observed in blood Cu levels in the probiotic yogurt group (*p* = 0.0004) but not in the conventional yogurt group (*p* = 0.9448) after 4 weeks of yogurt consumption; after 12 weeks, blood Cu levels decreased significantly in both groups (*p* < 0.0001) (Fig. 2a). Similarly, in the high- and medium-level sets, a significant decrease in blood Ni levels was observed earlier in the probiotic yogurt group than in the conventional yogurt group (Fig. 2b). However, in the low-level set, no significant decrease was noted in blood Cu or Ni levels of the two groups (Fig. 2a, b, *p*_Cu_ > 0.9999; *p*_Ni_ > 0.9999). A significant decrease in both blood Cu and Ni levels was observed within 4 weeks of probiotic yogurt consumption compared with conventional yogurt consumption. At the end of the trial, the blood Cu and Ni levels were found to have reduced by 34.45% (from 1246 ± 207.7 to 817.0 ± 145.1 μg/L) and 38.34% (from 4.855 ± 3.392 to 2.994 ± 1.942 μg/L), respectively, in the probiotic yogurt group, with both values being more significant than those noted in the conventional yogurt group. Similar results were obtained using urine samples (Fig. 2c). Conversely, the fecal samples showed elevated Cu and Ni levels compared with the baseline values (*p* < 0.0001 and *p* = 0.0003, respectively) after 12 weeks of the intervention, indicating increased HM excretion with yogurt ingestion (Fig. 2d).

With the elimination of the residual HM content from the body, improvements in some indicators associated with OS and inflammation in serum were evident in the probiotic yogurt group after 12 weeks of intervention; these indicators included CAT activity, anti-inflammatory IL-10 and IL-4 levels, and proinflammatory IL-6 and IL-1β levels (Fig. 2e–g; *p*_CAT_ < 0.0001, *p*_IL-10_ = 0.0066, and *p*_IL-4_ = 0.6205; *p*_IL-6_ = 0.0011 and *p*_IL-1β_ < 0.0001). No significant change in serum MDA levels was detected in either group (Fig. 2e), and normal liver and kidney function was observed after the intervention (Supplementary Table 5). Furthermore, the overall metabolic function of the GM of all participants was evaluated by measuring the level of short-chain fatty acids (SCFAs) in feces (such as acetate, *p* = 0.0322; propionate, *p* = 0.0061; and butyrate, *p* = 0.0469), which exhibited significant elevation in the probiotic yogurt group but not in the conventional yogurt group (Fig. 2h). The results of Spearman’s correlation analysis showed that the reduction in HM load with probiotic consumption correlated negatively with the increase in anti-inflammatory cytokine levels (IL-4 and IL-10) (*p* < 0.0001) and CAT activity (*p* < 0.0001) and positively with the decrease in OS biomarker level (MDA, *p* < 0.0001) (Supplementary Fig. 4).

After 4 weeks of initial yogurt consumption by the participants, *P. acidilactici* GR-1 was significantly enriched in their fecal samples (*p* < 0.0001, one-way analysis of variance [ANOVA] with Tukey’s post hoc analysis), indicating good adherence in the participants (Supplementary Fig. 5). After the completion of the trial, the relative abundance of GR-1 was significantly higher than the baseline value (*p* < 0.0001, Supplementary Fig. 5b), suggesting persistent colonization of GR-1 in the intestine.

### Reconstruction of GM by probiotics contributed to increased SCFA production and correlated with OS resistance

To investigate the effect of probiotic yogurt consumption on the GM of workers exposed to HMs, we analyzed the fecal samples of workers at baseline (low, medium and high-level set [LC, MC, and HC]) and study completion (LC, MC, and HC after probiotic yogurt treatment [PTLC, PTMC, and PTHC]) via 16S rRNA gene sequencing. We identified a total of 1514 amplicon sequence variants (ASVs) from 36 fecal samples. α-diversity showed no statistically significant differences among the six groups (Supplementary Fig. 6a**;** *p*_Chao_ = 0.6273 and *p*_Shannon_ = 0.5158). Principal component ordination analysis (PCoA) (Supplementary Fig. 6b, c) showed that the ASV of the three subsets (LC, MC, and HC) were grouped at enrollment but separated into two groups after the intervention, suggesting that the variability in participants was increased.

**Fig. 3:**
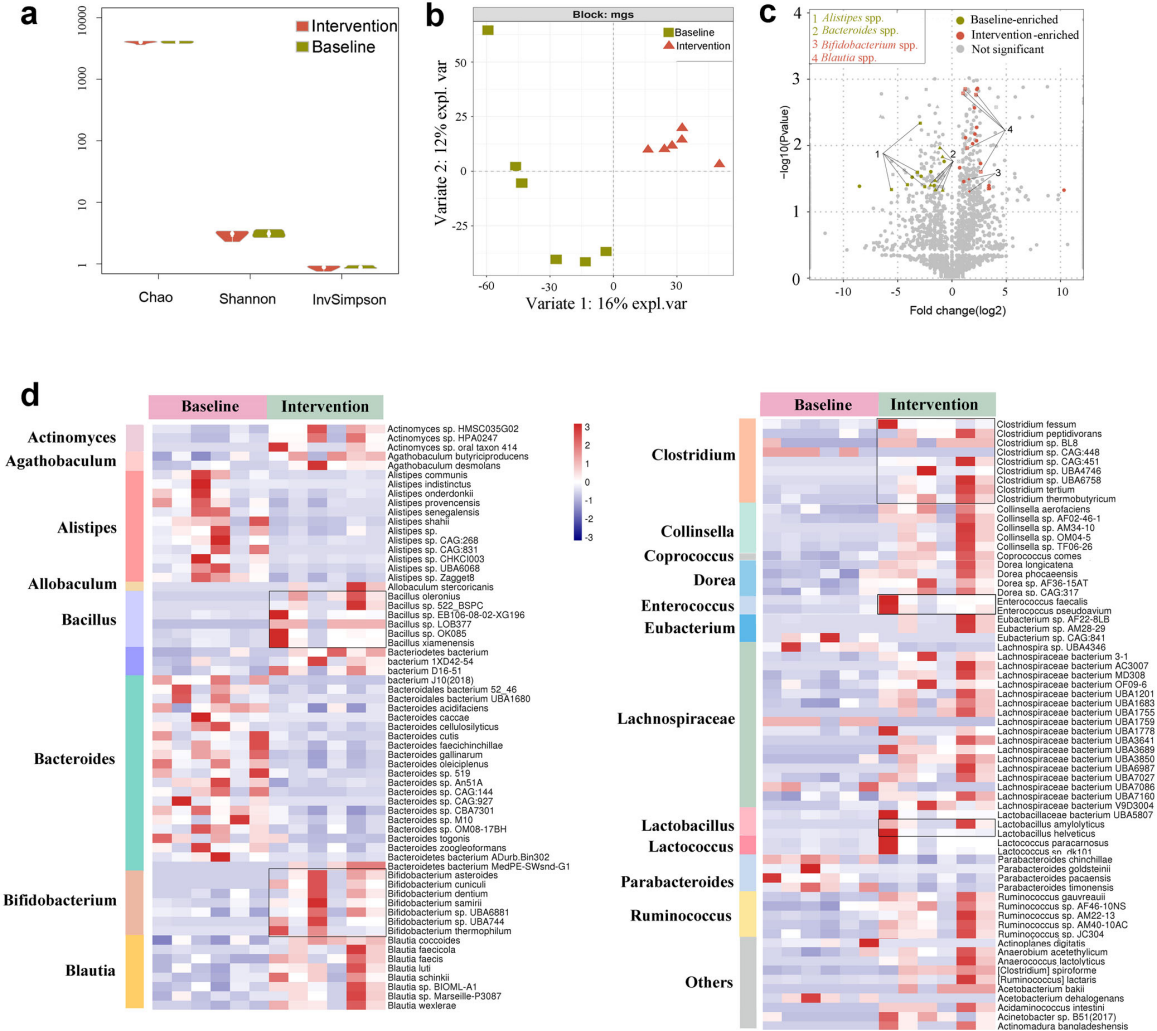
Probiotic yogurt significantly altered the symbiosis of gut microbiota after 12 weeks of intervention. **a** No significant differences were noted in bacterial α-diversity values between the two groups. **b** Bacterial signatures between the two groups varied significantly (Bray–Curtis distance, PERMANOVA, *p* = 0.025), as shown using DIABLO. **c** Signatures of gut microbiota in the baseline and intervention groups. The x-axis shows the fold change of a species abundance of the two groups. The y-axis shows the −log10 (*p* value) of each species in the two groups. Species with significant difference in abundance between the two groups are shown in green (baseline-enriched) and red (intervention-enriched); species belonging to the same genus are linked by lines. Species with no significant difference in abundance between the two groups are shown in gray. Metastats was used to determine MGS signatures. **d** The heatmap shows the prominent species that differed significantly in abundance between the baseline and intervention groups. Red indicates enrichment and blue decrease. The magnitude of the changes is proportional to the color intensity. *n* = 6 in each group. Wilcoxon rank-sum test was performed. Source data and exact *p* values are provided in the source data file.

After identifying similar taxa at baseline and distinct taxa after yogurt intervention via 16S rRNA gene sequencing, we intended to increase the resolution of these findings through metagenome sequencing of six random samples at baseline and the end of the trial. The information regarding the six random samples is presented in Supplementary Tables 6 and 7. On average, 92.12 million reads (12 GB data) were generated per fecal sample; a gene catalog of 11.4 million nonredundant genes was assembled. The genes were annotated to 4155 Kyoto Encyclopedia of Genes and Genomes (KEGG) functional categories and organized into 13,945 metagenomic species (MGS). The overall community composition was consistent with the 16S rRNA sequencing profiles. The consumption of probiotic yogurt exerted no significant effect on the α-diversity indices such as Richness, Chao1, Shannon, and Simpson indices (Fig. 3a); nonetheless, it modulated the structure of GM, according to the result of the DIABLO sample plot (Fig. 3b**;** *p* = 0.025, permutational multivariate ANOVA [PERMANOVA] of Bray–Curtis distances). At the phylum level, Firmicutes (*p* = 0.1620) and Actinobacteria (*p* = 0.2274) were enriched in the worker group, whereas Bacteroidetes (*p* = 0.1142) was depleted (Supplementary Fig. 7). The species enriched in the worker group after yogurt consumption included SCFA-producing bacteria such as *Agathobaculum* spp.^47^ (*A. butyriciproducens* and *A. desmolans*), *Allobacterium* spp.^48^, *Blautia* spp.^49^ (*Blautia faecis*, *Blautia wexlerae*, *Blautia luti*, *Blautia obeum*, and *Blautia faecicola*), *Bifidobacterium* spp.^50^ (primarily *B*. *ruminantium* and *B. adolescentis*), *Clostridium* spp.^51^, *Collinsella* spp. (primarily *C. aerofaciens*^52^), *Enterococcus* spp. (*E. faecalis*^53^), *Lachnospiraceae* bacterium spp.^48^, and *Ruminococcus* spp.^51^, and *Coprococcus comes*^54^. The depleted species included *Alistipes* (*A. shahii*, *A. senegalensis*, *A. communis*, *A. onderdonkii*, and *A. ihumii*) and *Bacteroides* (*Bac. caecimuris*, *Bac. ovatus*, *Bac. cutis*, *Bac. finegoldii*, and *Bac. acidifaciens*) (Fig. 3c, d). Therefore, the increase in the fecal levels of SCFAs noted in the worker group after yogurt consumption might have resulted from the enrichment of potential SCFA-producing bacteria.

At the module or pathway level, the GM of the worker group that consumed probiotic yogurt was enriched in KEGG orthologs involved in OS response, including superoxide dismutase (K04565 and K04564) (*p* = 0.0411), CAT (K03781) (*p* = 0.5887), glutathione peroxidase (K00432) (*p* = 0.3095), and glutathione reductase (NADPH) (K00383) (*p* = 0.2406) (Supplementary Table 8). This result suggested that probiotic yogurt induces extensive bacterial defense mechanisms against HM-induced OS. In addition, the GM functions essential for glycolysis/gluconeogenesis (*p* = 0.00388), quorum sensing (*p* = 0.00395), purine metabolism (*p* = 0.00395), and ABC transporters (*p* = 0.00209) were enhanced (Supplementary Fig. 8).

### Probiotic yogurt altered fecal and serum metabolomes of the worker group

**Fig. 4:**
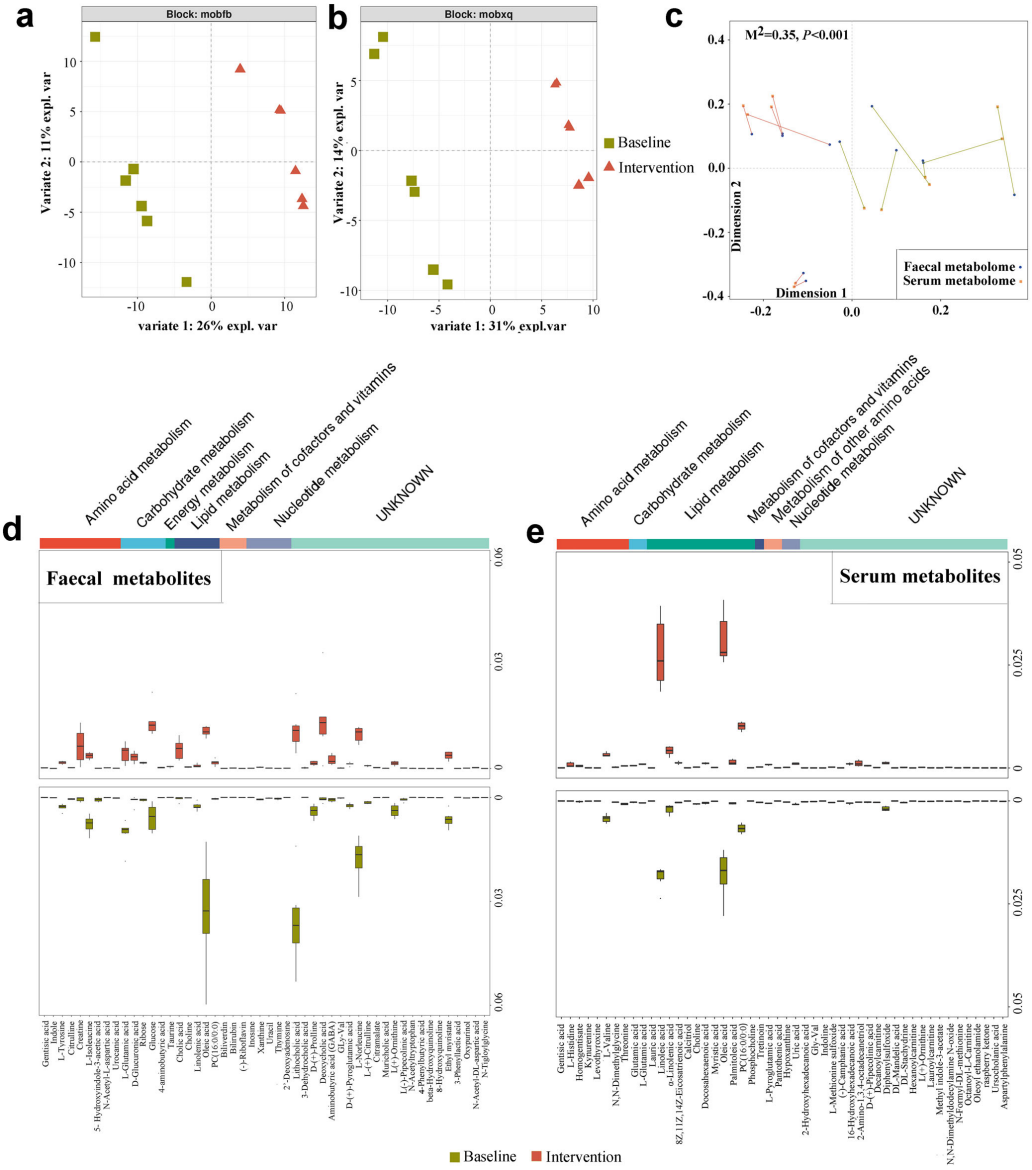
Probiotic yogurt altered fecal and serum metabolomes after 12 weeks of intervention. DIABLO sample plots comparing the fecal and serum metabolites before and after yogurt intervention. **a** Separation of fecal metabolome between the baseline and intervention groups after 12 weeks of probiotic yogurt intervention, as shown using DIABLO. *n* = 6 in each group. **b** Separation of serum metabolome between the baseline and intervention groups after 12 weeks of probiotic yogurt intervention, as shown using DIABLO. *n* = 6 in each group. Each point in figures **a** and **b** represents an individual sample. **c** Procrustes analysis of fecal metabolome versus serum metabolome. Fecal and serum samples are shown as blue and orange dots, respectively; fecal and serum samples from the same individuals are connected by red (baseline) and cyan (intervention) lines. The top 50 differential metabolites (**d**) and differential serum metabolites (**e**) between groups before and after probiotic yogurt intervention. The differential abundance of fecal and serum metabolites between the two groups was evaluated using Student’s *t* test.

We analyzed the fecal and serum metabolites associated with GM via liquid chromatography (LC)–mass spectrometry (MS). The baseline and postintervention metabolomes were found to vary significantly (Fig. 4a, b). Compared with baseline, a total of 99 differential fecal metabolites (40 downregulated and 59 upregulated) were detected after 12 weeks of probiotic yogurt intervention (Supplementary Table 9). Specifically, the levels of metabolites associated with antioxidation, including amino acid (creatine, *p* = 0.0365; gentisic acid, *p* = 0.0134; and γ-aminobutyric acid, *p* = 0.0291), lipid (choline, *p* = 0.0023), nucleotide (inosine, *p* = 0.0353), energy (taurine, *p* = 0.0004), and bile acid (cholic acid, *p* = 0.0251; 3-dehydrocholic acid, *p* = 0.0143; and deoxycholic acid, *p* = 0.0368) metabolisms, were elevated in postintervention fecal samples, whereas indole (*p* = 0.0243), proline (*p* = 0.0209), tyrosine (*p* = 0.0225), and lithocholic acid (*p* = 0.0019) levels were markedly reduced (Fig. 4d). Regarding the serum metabolome, a total of 76 (28 downregulated and 48 upregulated) metabolites exhibited various levels (Supplementary Table 10). The serum metabolome after intervention was characterized primarily by the enrichment of metabolites involved in antioxidation, such as pantothenic acid (*p* = 0.0176), α-linolenic acid (*p* = 0.0087), L-glutamic acid (*p* < 0.0001), pyroglutamic acid (*p* = 0.0002), histidine (*p* = 0.0162), and deoxycholic acid (*p* = 0.0397) as well as depletion of metabolites involved in OS, such as methionine sulfoxide (*p* = 0.0009), hypoxanthine (*p* = 0.0419), and kynurenine (*p* = 0.0333) (Fig. 4e). Together, probiotic yogurt intervention appeared to be the primary contributor to the enhancement of antioxidant activities between the pre- and postintervention metabolomes in both feces and serum samples. Moreover, Procrustes analysis^55^ revealed strong co-operativity between fecal and serum metabolome profiles (Fig. 4c; *p* < 0.001), suggesting that the metabolic alterations in the GM of the worker group were associated with the changes in serum metabolite profiles.

Metabolic pathway enrichment analysis was performed using the differential metabolites. The results showed that the metabolic pathways in fecal and serum metabolomes, including histidine, α-linolenic acid, and D-glutamine and D-glutamate metabolisms, were enriched after the probiotic yogurt intervention (Supplementary Fig. 8). Specifically, ascorbate and aldarate metabolism and taurine and hypotaurine metabolism were enriched in the fecal metabolome and glutathione metabolism and pantothenate and CoA biosynthesis were enriched in the serum metabolome (Supplementary Fig. 9).

### Microbiome alterations correlated with metabolome changes in the worker group

**Fig. 5:**
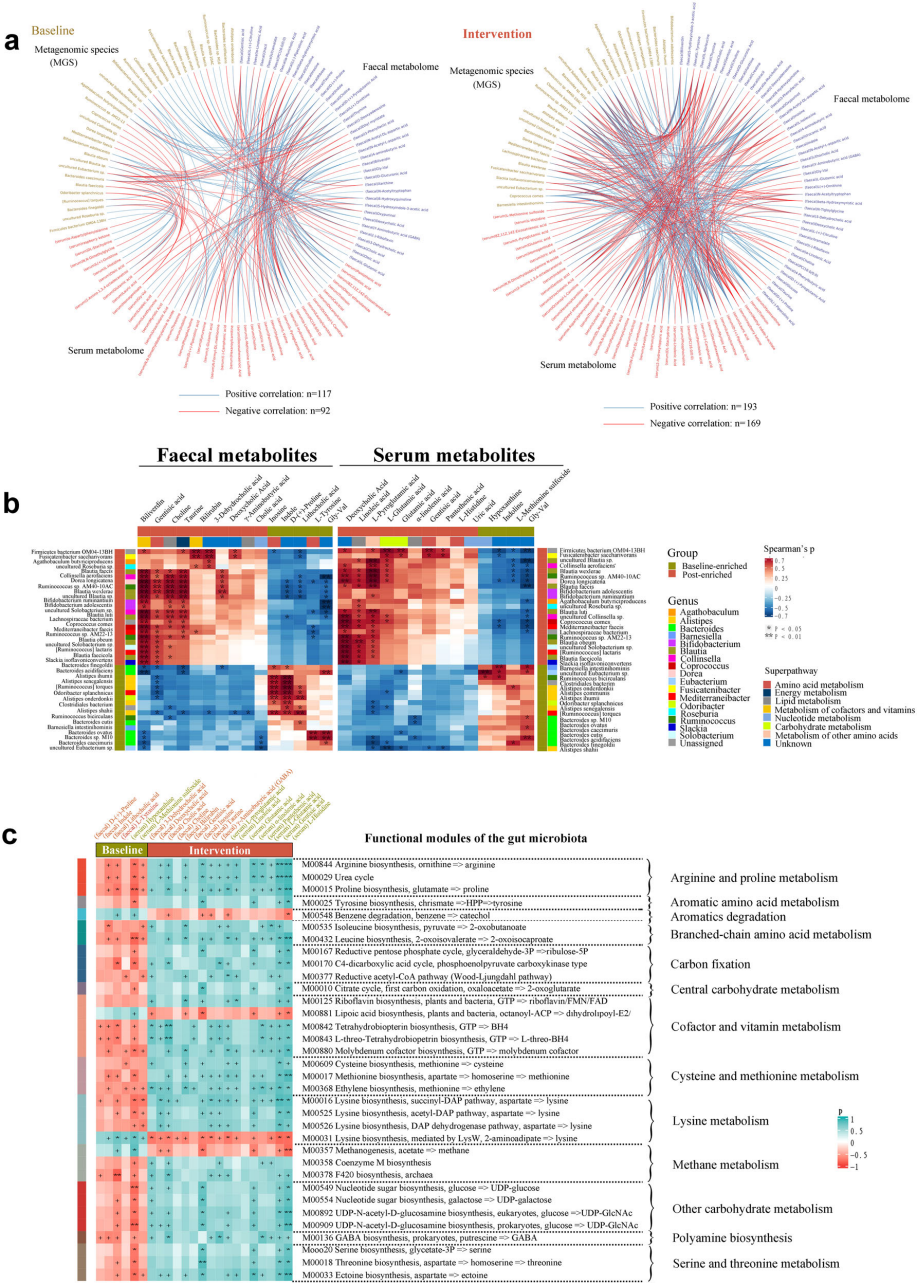
Gut microbiota influenced host fecal and serum metabolomes in the worker group after 12 weeks of probiotic consumption. **a** The interomic correlation networks of all variables for the gut microbiota and fecal and serum metabolomes of the workers at baseline and after the probiotic yogurt intervention. **b** Correlation of members of the differential MGS with fecal or serum metabolites associated with OS or antioxidation. Species that varied significantly between the two groups were identified using Metastats. Enrichment in either group is indicated using colored bars at the left and top of the plot. Significant correlations are denoted using stars (*p < 0.05, **p < 0.01, Student’s *t* test), and Benjamini–Hochberg adjustment was performed for multiple comparisons. In the heatmap, the legend denotes correspondingly the R value of Spearman. Exact *p* values are provided in the source data file. **c** Correlation of the levels of baseline-enriched (relevant to OS) and intervention-enriched metabolites (relevant to antioxidation) with microbial functions. The heatmap shows the Spearman’s correlation coefficients between fecal (red text) or serum (green text) metabolites and functional modules of GM. Significant correlations are denoted as +*p* < 0.05, *p < 0.01, and **p < 0.001. Source data and exact *p* values are provided in the source data file.

To further analyze the association between GM and fecal and serum metabolomes, we performed interomic correlation analysis. We detected close associations in samples both at baseline and study completion (Fig. 5a). Each of the enriched antioxidation-related metabolites correlated positively with most of the enriched species in the worker group after the yogurt intervention (Fig. 5b; Spearman’s correlation analysis), suggesting that the increase in antioxidation activities was related to the enrichment of specific species. Furthermore, some functional modules of GM (amino acid and carbohydrate metabolisms) significantly covaried with the levels of their antioxidation-related metabolites in both feces and serum samples (Fig. 5c), implying that energy availability was increased in the worker group after yogurt consumption. Moreover, the most enriched MGS and serum metabolites correlated negatively with blood Cu levels, whereas the most enriched MGS and fecal metabolites correlated positively with fecal Cu levels (Supplementary Fig. 10), suggesting that microbiota composition and host metabolism are responsive to the extent of Cu exposure.

**Fig. 6:**
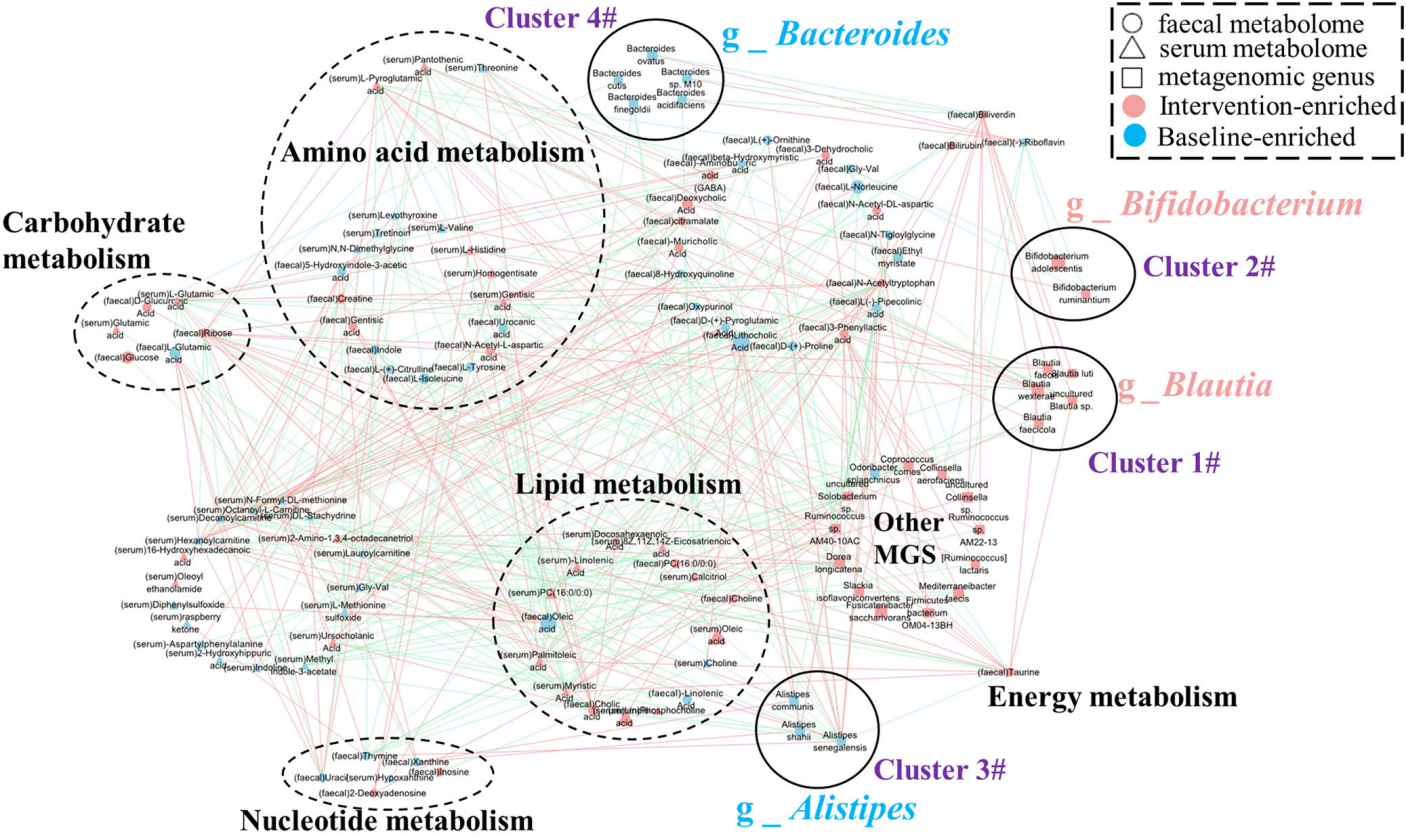
Co-occurrence network constructed based on the relative abundance of differential MGS, fecal metabolites, and serum metabolites of workers administered with probiotic yogurt. The differential MGS primarily generated four covarying units (clusters 1–4). The size of the nodes represents the abundance of these variables. Red and blue dots, respectively, indicate the increase and decrease of relative abundances of variables after the probiotic yogurt intervention compared with the baseline values. The edges between nodes indicate Spearman’s positive (red) or negative (blue) correlation; the edge thickness indicates the range of *p* values (*p* < 0.05). Source data and exact *p* values are provided in the source data file.

We further explored the effects of differential MGS on fecal and host metabolic functions. Co-occurrence analysis revealed that MGS had a close and extensive co-occurring relationship with both fecal and serum metabolites (Fig. 6). In the expression network, the differential MGS primarily generated four covarying clusters (clusters 1–4). Cluster 1 comprised five enriched species belonging to the genus *Blautia* (*Blautia faecis*, *Blautia luti*, *Blautia wexlerae*, *Blautia faecicola*, and uncultured *Blautia* sp.) after the intervention compared with the baseline data. Cluster 2 comprised two enriched species belonging to the genus *Bifidobacterium* (*Bifidobacterium adolescentis* and *Bifidobacterium ruminantium*). Cluster 3 comprised three depleted species belonging to the genus *Alistipes* (*Alistipes communis*, *Alistipes shahii*, and *Alistipes senegalensis*). Clusters 4 comprised five depleted species belonging to the genus *Bacteroides* (*Bacteroides ovatus*, *Bacteroides cutis*, *Bacteroides finegoldii*, *Bacteroides acidifaciens*, and *Bacteroides* sp. M10). In this network, the differentially altered fecal and serum metabolites were primarily involved in amino acid, lipid, carbohydrate, and nucleotide metabolisms. Some bacterial species within cluster 1 correlated positively with carbohydrate (*Blautia wexlerae* and *Blautia faecis*), amino acid (*Blautia faecis*), energy (*Blautia wexlerae* and uncultured *Blautia* sp.), and lipid (uncultured *Blautia* sp.) metabolisms. In contrast, all members of cluster 3 (*Alistipes communis*, *Alistipes shahii*, and *Alistipes senegalensis*) correlated positively with nucleotide metabolism; some members of clusters 3 and 4 (e.g., *Alistipes shahii*, *Alistipes senegalensis*, *Bacteroides finegoldii*, and *Bacteroides acidifaciens*) correlated negatively with lipid metabolism. The other enriched MGS in the intervention group also correlated positively with amino acid (e.g., *Ruminococcus* sp. AM22-13, *Ruminococcus lactaris*, *Ruminococcus* sp. AM40-10AC, and *Dorea longicatena*), carbohydrate (e.g., *Ruminococcus* sp. AM40-10AC and *Dorea longicatena*), and lipid (e.g., *Dorea longicatena*, *Collinsella* sp., *Solobacterium* sp., and *Ruminococcus* sp. AM40-10AC) metabolisms. These results suggested that these key differential MGS exerted a synergistic effect on the workers who consumed probiotic yogurt that influenced fecal and host metabolism.

### GM integrity is essential for probiotics to reduce Cu accumulation in mice

To assess whether the integrity of GM was essential for HM remediation, the GM of mice was disrupted through the oral administration of antibiotics for 10 days before Cu exposure. All mice treated with antibiotics had approximately half of intestinal bacterial load remaining, as indicated by the decreased number of culturable gut microbes (Supplementary Fig. 11a, b; *p* < 0.0001, Wilcoxon rank-sum test) and lower copy numbers of 16 S rRNA genes in feces (Supplementary Fig. 11c; *p* = 0.0053). The taxonomic composition of antibiotic-treated GM was significantly disrupted compared with that of the untreated control, with few overlapping operational taxonomic units, a significant decrease in the relative abundance of the major phyla (*p* < 0.0001, one-way ANOVA with Tukey’s post hoc analysis), and an evident decrease in the abundance of the types of genera in the antibiotic-treated GM compared with that in the untreated control GM (Supplementary Fig. 11d–f).

**Fig. 7:**
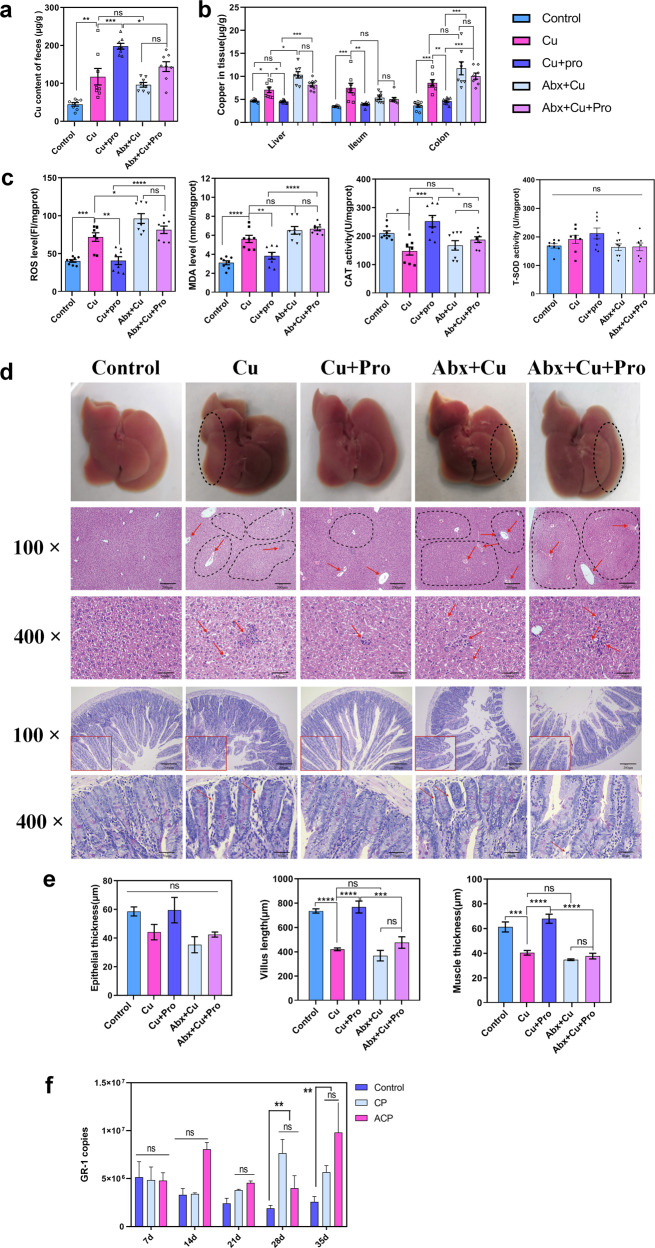
Gut microbiota was essential for the probiotic strain GR-1 to alleviate Cu toxicity in mice. Cu levels in the feces (**a**) and tissue (**b**) samples of mice (*n* = 8 biologically independent animals). **c** The levels of ROS and MDA and activity of CAT and total SOD in the mouse liver sample (*n* = 8 biologically independent animals). **d** Representative images of hematoxylin and eosin–stained liver sections and Alcian blue and periodic acid–Schiff base–stained small intestinal sections (*n* = 3 biologically independent animals). The area within the solid square in 100× images of the small intestine has been magnified to 400×. Scale bars: 200 μm in 100× images and 50 μm in 400× images. The arrows and area circled using the dotted line indicate the liver damage. The liver tissue of the control exhibiting normal appearance; the liver tissue of the mice belonging to the Cu, Abx + Cu, and Abx + Cu + Pro groups showing cytoplasmic vacuolization, chromatin condensation, and pyknotic nuclei; the liver tissue of the mice belonging to the Cu+Pro group exhibiting preserved liver appearance, alleviated cytoplasmic vacuolization, and pyknotic nuclei. **e** Intergroup comparison of the averages of epithelial thickness, villus length, and muscle thickness of the ileum (*n* = 3 biologically independent animals). **f** Absolute copies of GR-1 in the feces sample of mice that were orally administered with GR-1 (*n* = 3 biologically independent animals). Data are expressed as means ± standard errors of the means. Statistical analysis was performed using one-way ANOVA with Tukey’s post hoc analysis using GraphPad Prism (version 8.0.1) (*p < 0.05, **p < 0.01, ***p < 0.001, and ****p < 0.0001, ns represents not significant). Source data are provided as a source data file.

(1) Oral gavage of CuSO_4_ solution resulted in significantly more Cu excretion in the feces and more accumulation in the tissues than the control (Fig. 7a, b; *p*_feces_ = 0.0017, *p*_liver_ = 0.0259, *p*_ileum_ = 0.0003, and *p*_colon_ = 0.0003). OS in the liver was increased, and the liver (cytoplasmic vacuolization, chromatin condensation, and pyknotic nuclei) and small intestine (shorter villus length, *p* < 0.0001; thinner muscle, *p* = 0.0003) exhibited evident damages after Cu exposure (Fig. 7c–e). (2) Cu-induced damages were exacerbated in the Abx+ Cu group, which resulted in the disruption of GM. We detected 18.13% less fecal Cu excretion, 45.7% more Cu accumulation in the liver (*p* = 0.0108), and 36.8% more Cu accumulation in the colon (*p* = 0.0674) (Fig. 7a, b). Furthermore, OS (*p*_ROS_ = 0.0135; *p*_MDA_ = 0.2897) in the liver was more severe than that in the Cu group (Fig. 7c–e). (3) Administration of the probiotic strain GR-1 to Cu-exposed mice increased the fecal excretion of Cu by 68.07% (*p* = 0.0005) and accordingly decreased the tissue accumulation of Cu (35.83% less in the liver, *p*_liver_ = 0.00164; 47.33% in the ileum, *p*_ileum_ = 0.0017; and 45.98% in the colon, *p*_colon_ = 0.0036) (Fig. 7a, b) compared with those in the Cu exposure group. The OS (*p*_ROS_ = 0.001, *p*_MDA_ = 0.0034, and *p*_CAT_ = 0.0001) and tissue damage were significantly alleviated, as indicated by less cytoplasmic vacuolization in the liver and improvement of the intestinal mucosa morphology (Fig. 7c–e; shorter villus length, (*p* < 0.0001; thinner muscle, *p* < 0.0001). (4) However, the remediating effect of GR-1 was reduced in GM-disrupted mice as there were no significant differences in residual Cu levels in tissues between the Abx + Cu + Pro and Abx + Cu groups (Fig. 7a, b; *p*_liver_ = 0.2434, *p*_ileum_ = 0.9935, and *p*_colon_ = 0.5423). Concomitantly, no obvious mitigative effect of GR-1 on Cu toxicity was observed in the GM-disrupted mice (Fig. 7c–e; *p*_ROS_ = 0.2520, *p*_MDA_ = 0.9967, and *p*_CAT_ = 0.8576; shorter villus length, *p* = 0.2804; thinner muscle, *p* = 0.9430). To rule out the colonization difference of GR-1 between the groups, we performed quantified real time (RT)-polymerase chain reaction (PCR); the results revealed no significant difference in the absolute number of GR-1 between mice that received and those that did not receive antibiotic treatment. This finding indicates that the abundance of GR-1 is not a major factor influencing HM remediation (Fig. 7f). To summarize, the antibiotic treatment demonstrated that the integrity of indigenous GM is essential for the protective effects of GR-1 against Cu toxicity in mice.

### Probiotic GR-1 maintains the stability of the redox state and structural composition of GM in mice

**Fig. 8:**
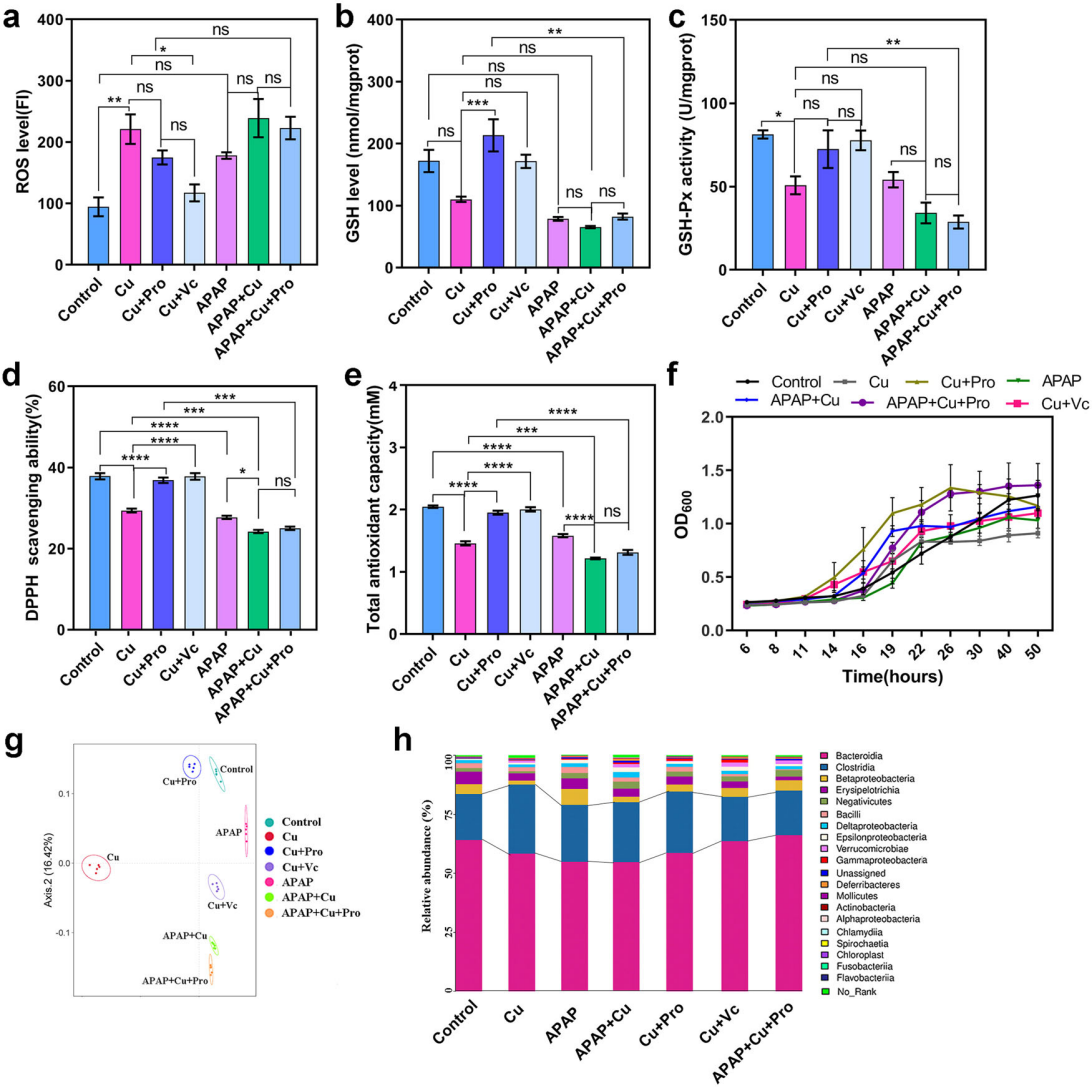
Probiotic strain GR-1 alleviated OS and altered the composition of GM in mice. The OS status (**a** ROS level, **b** GSH level, and **c** GSH-Px activity) and antioxidant activity (**d** DPPH free radical**–**scavenging ability and **e** the total antioxidant capacity) of intestinal contents with different treatments. **f** The growth curve of GM in BGM growth medium containing 50 mg/L Cu under anaerobic condition. **g** PCA analysis of β-diversity in GM structures among the seven groups based on the Bray–Curtis distance. **h** The average relative abundances of GM at the class level. Data are expressed as means ± standard errors of the means in figure **a**–**f**. *n* = 3 per group in figure **a**–**f** and *n* = 5 per group in figure **g** and **h**. **a**–**e** statistical analysis was performed using one-way ANOVA with Tukey’s post hoc analysis (*p < 0.05, **p < 0.01, ***p < 0.001, and ****p < 0.0001, ns represents not significant) using GraphPad Prism (version 8.0.1). Source data are provided as a source data file.

OS is a key feature of HM toxicity^56^. Paracetamol (acetaminophen [APAP]) is a common analgesic and antipyretic drug that can induce OS and result in glutathione (GSH) diminution when administered at high doses^57^. The probiotic GR-1 exhibited a strong Cu and APAP resistance as well as antioxidant capacity in vitro (Supplementary Fig. 12). Therefore, we investigated the possible role of the antioxidant capacity of GR-1 in maintaining the intestinal redox homeostasis and thereby the normal function of GM. (1) Similar to Cu, APAP administration resulted in elevated OS in the gut, as evidenced by increased levels of ROS (*p*_Cu_ = 0.0044 and *p*_APAP_ = 0.0207) as well as decreased activities of GSH (*p*_Cu_ = 0.0520 and *p*_APAP_ = 0.0023) and GSH peroxidase (GSH-Px) (*p*_Cu_ = 0.0467 and *p*_APAP_ = 0.0906) in the intestinal contents (Fig. 8a–c). (2) Both GR-1 (*p*_ROS_ = 0.6074, *p*_GSH_ = 0.0009, and *p*_GSH-Px_ = 0.2417) and vitamin C (Vc) (*p*_ROS_ = 0.3680, *p*_GSH_ = 0.0550, and *p*_GSH-Px_ = 0.0951) supplementation remarkably or partially reversed the changes induced by Cu. In addition, the antioxidant capacity and Cu tolerance of the GM of Cu-exposed mice were recovered after treatment with GR-1 or Vc (Fig. 8d–f; *p*_2,2-diphenyl-1-picryl-hydrazyl-hydrate [DPPH]_ < 0.0001 and *p*_total antioxidant capacity [TAC]_ < 0.0001), suggesting that the antioxidant defense system of GM under OS was protected in the presence of the probiotic GR-1. (3) The OS level in the intestinal tract was further elevated by simultaneous exposure to Cu and APAP; however, GR-1 could no longer exert its effect on the maintenance of the redox homeostasis, suggesting that OS had exceeded the antioxidant capacity of GR-1 (Fig. 8b–f). Correspondingly, the antioxidant capacity and Cu tolerance of the GM of APAP- and Cu-exposed mice could not be recovered (Fig. 8d–f). The protective effect of GR-1 on GM was further confirmed by comparing the compositional structures of microbiota in different groups. PCA revealed that the probiotic remediation group (Cu + Pro) showed the highest similarity in microbiota structure to that of the control group but was different from the other groups (Fig. 8h). The GM of Cu, APAP, and APAP + Cu groups showed similar changes in community composition, which were partially reversed by probiotic or Vc administration (Fig. 8i). Together, these results demonstrate that GR-1 mitigates Cu toxicity by alleviating OS and protecting GM function.

## Discussion

The application of probiotics, as a therapeutic strategy to remediate HM toxicity in humans, has great potential. Here we present and extend upon work in this area to better elucidate the beneficial impacts of probiotics for wider applications^24–26^. Probiotic yogurt fermented with *P. acidilactici* GR-1 was found to efficiently reduce toxic metal residues in this randomized controlled human trial. Experiments performed in mice demonstrated that the probiotic GR-1 exerted protective effects on microbiota composition and redox stability, mitigating the harmful effects of HM exposure.

Heavy metal toxicity if often underestimated in human populations across the globe^26,43,58^. Currently, epidemiological studies of HM toxicity in developing countries are limited, likely leading to the under-reporting of this public-health issue^3^. In the present study, the blood Cu and Ni levels in occupational workers were significantly reduced by 34.45% and 38.34%, respectively, after 12 weeks of probiotic yogurt consumption. The HM remediation effect of GR-1-enriched yogurt was similar to that observed in a previous human trial using lyophilized *L. plantarum* CCFM8610 to remove Cd residues^27^ but superior to the result obtained in a previous report using yogurt containing nonspecific probiotics^26^. These results suggested that the HM-lowering effect of a probiotic depends less on its carrier form but more on its functionality. GR-1 is a probiotic strain with specific characteristics of strong HM resistance and antioxidant capacity, and can easily be incorporated, with yogurt, into probiotic capsules. Yogurt serves as an effective medium to maintain the viability of a probiotic for short periods^59,60^. Results also demonstrated the ability of probiotic yogurt to protect the host against the deleterious effects of HMs as it improved the HM-induced alteration of serum indices.

The GM-modifying effect of probiotics under HM stress has been investigated extensively in animals^2,36^, although rarely in humans. In the participants of this study, the composition, rather than the overall diversity of GM, was altered significantly by GR-1^61^. The abundance of the phylum Proteobacteria, an indicator of gut dysbiosis, markedly decreased with yogurt intervention, suggesting a remission in proinflammatory activities both locally and systemically^62–64^. The phylum Firmicutes, largely comprising gram-positive species, showed increased abundance after yogurt intervention. Gram-positive bacteria such as *Bacillus* sp., *Clostridium* sp., *Enterococcus* sp., *Lactobacillus* sp., and *Bifidobacterium* sp. belonging to the Actinobacteria group possess high metal-adsorptive and metal-removal capacities because of high peptidoglycan and teichoic acid contents in their cell walls^65–67^. Hence, the enrichment of MGS belonging to these genera suggested the increased potential of GM to excrete Cu and Ni. Furthermore, the increased abundance of genera such as *Allobaculum*, *Lactobacillus*^68^, and *Bifidobacterium*^69^ may correlate with enhanced gut barrier function and decreased gut permeability. These findings are consistent with those of previous studies^29,70^. We analyzed the GM dynamics of the workers who consumed the probiotic yogurt but not that of the workers and students who consumed the conventional yogurt; this further allowed us to delineate the state of dysbiosis of the workers who consumed the probiotic yogurt and its correlation with HMs and other physiological parameters. In the future, the sequencing of GM genome obtained from a large cohort may provide insights into the detailed action mechanism of probiotics, for example, the specificity of probiotic action at the microbiome level.

The metagenomic analysis performed in this study showed that probiotic-enriched species primarily belonged to the genera *Blautia*, *Bifidobacterium*, *Lachnospiraceae*, and *Lactobacillus* and the depleted species belonged to the genera *Bacteroides*, *Alistipes*, and *Parabacteroides*. Among them, *Bifidobacterium* and *Lactobacillus* are two representative bacteria of the human GM that have antioxidant capacity^71^. *Alistipes*, *Bacteroides*, and *Parabacteroides* correlated negatively with superoxide dismutase (SOD) level and positively with OS level; in contrast, unidentified *Lachnospiraceae* and *Lactobacillus* correlated positively with SOD level and negatively with ROS level^72^, suggesting the amelioration of OS in the GM by probiotics. *Blautia*^73,74^ and other SCFA (particularly butyric acid)-producing bacteria such as *Collinsella aerofaciens*^52^, *Coprococcus comes*^54^, and *Agathobaculum butyriciproducens*^47^ have also been reported to be enriched after probiotic administration. SCFAs regulate intestinal inflammation, enhance intestinal barrier function, and reduce OS^75^. The enrichment of SCFA-producing bacteria may contribute to the reduction of HM-induced stress in the intestine. The abundance of several indole-positive species such as *A. shahii*, *A. senegalensis*, and *A. onderdonkii*^76^ decreased after probiotic administration, implying reduced infections caused by enteric bacteria, as indole production is a vital phenotypic characteristic of bacteria that has long been used to differentiate, identify, and diagnose enteric bacterial infections^77^.

Fecal metabolomics data provided further evidence for enhancement of the antioxidant and metal-removal capacities of GM. For instance, yogurt consumption resulted in increased levels of gentisic acid, which has antioxidant capacity and scavenges free radicals in vitro and in pulse radiolysis studies^78^. Elevated levels of another metabolite, taurine, reportedly exert protective and detoxification effects on Cr (VI)-induced OS in mice and antioxidant restoration in rats^79,80^. However, the fecal indole levels in the worker group that consumed yogurt were decreased, probably because of a decrease in the abundance of indole-positive species. Probiotic yogurt supplementation also significantly increased the fecal secretion of bile acid, which may correlate with the enhanced fecal excretion of Cu and Ni. Some commensal bacteria such as *Lactobacillus*, *Bifidobacterium*^81^, and *Ruminococcus*^82^ can increase HM excretion in mice by modulating bile acid metabolism^58^.

GM may significantly shape host metabolism^83^. In this study, we detected profound alterations in serum metabolic profiles and pathways in the worker group after probiotic intervention. Elevated levels of serum metabolites and pathways are primarily associated with increased antioxidant defense, such as the metabolite of α-linolenic acid^84^, histidine^85^, pantothenic acid^86^, L-glutamic acid^87^, pyroglutamic acid, histidine^88^, and the metabolic pathway of D-glutamine and D-glutamate^89^. Similarly, α-linolenic acid is associated with protection against OS in rats challenged with organic mercury^84^; histidine accumulation is a normal process used for diminishing the toxicity of Cu and some other HMs^85^. On the other hand, some metabolites were significantly reduced after probiotic yogurt intervention. For instance, methionine sulfoxide—a product of methionine oxidation via ROS—is considered a biomarker of OS in vivo^90^. Plasma hypoxanthine and kynurenine levels have been reported as OS-related implications^91,92^. Together, corresponding metabolic changes in humans contribute to reduced OS and inflammation in the intestine, which might have result in the decreased serum levels of MDA, IL-1β, and IL-6 as well as the increased serum levels of CAT, IL-4, and IL-10 in the worker group after the probiotic yogurt intervention.

Studies using germ-free and gnotobiotic mice have demonstrated the essential role of GM integrity in limiting HM uptake^31,32^. The mouse experiments performed in this study demonstrated that dysbiosis of GM, in which the microbiota was disrupted but not depleted by the antibiotic cocktail, also affected both the uptake of Cu and the detoxification effect of GR-1. Vc is a basic compound that is essential for antioxidation^93^. Therefore, we compared the remediating effect of GR-1 and Vc; they exerted similar effects in alleviating Cu-induced OS in mice (Fig. 8), which supports the fact that the antioxidant capacity of probiotics is the primary mechanism underlying the improvement of HM-induced toxicity^94,95^. Although promising results have been achieved with Vc, it cannot continue to function in the body once its administration has been stopped as it is not stored in the body^93^. Furthermore, Vc did not exert any modulating effect on Cu-induced GM dysbiosis. However, the probiotic GR-1 colonized in the intestine and exhibited a long-term role in both the amelioration of Cu toxicity and maintenance of GM homeostasis in mice (Fig. 8a–e)^2^. To enhance the remediating effect of probiotics, their coadministration with prebiotics may be considered for future applications because prebiotics are significant health promoters^96^.

Based on the present and previous studies, it seems reasonable to use probiotics with strong antioxidant capacity to combat HM toxicity. Considering the wide range of people at risk of HM exposure, probiotic-based gut remediation is a feasible and promising intervention strategy for the detoxification of HMs.

## Methods

### Design of the study and collection of human samples

This randomized, double-blind, clinical trial was approved by the Health Sciences Research ethics board at The First Hospital of Lanzhou University, Gansu, China (ClinicalTrials.gov identifier: ChiCTR2100053222), and the experiments were performed according to approved guidelines. Participants were enrolled between July 17, 2020, and August 27, 2020. All participants provided signed informed consent before enrollment. The exclusion criteria were as follows: (1) use of antibiotics within 1 month before study commencement; (2) regular use of probiotic supplements; (3) lactose intolerance; (4) alcohol or drug abuse; (5) presence of severe comorbidities such as kidney disease, liver disease, inflammatory intestinal disease, thyroid disorder, or immunodeficiency disease and any medicine use. Based on the human trials conducted by Sadrzadeh-Yeganeh et al.^97^ and Ejtahed et al.^98^, we set the number of participants in this study at ≥30 per group. Two independent cohorts were created: 30 college students from the School of Life Sciences, Lanzhou University (Gansu, China), as the control cohort and 152 occupational workers from a metallurgical enterprise in Jinchang city (Gansu, China) as the intervention cohort. Then, the participants from the worker cohort were randomly categorized into two groups—the probiotic yogurt and conventional yogurt groups— in a 1:1 ratio. The yogurts consumed by both groups contained *Lactobacillus bulgaricus* and *Streptococcus thermophilus*. In addition, the probiotic yogurt contained *P. acidilactici* GR-1, a potent strain with demonstrated Cu resistance (Supplementary Fig. 2). Over a period of 12 weeks, the participants were instructed to consume 250 g of yogurt daily (which contained at least 1 × 10^10^ CFU/250 g of each bacterial strain). This dose was decided based on a previous study on probiotic yogurt^26^. The yogurt products were freshly prepared every 7 days during the trial and distributed to the participants by a local dairy manufacturer. Viability of the probiotics in each batch was evaluated in the laboratory (Supplementary Fig. 3). Both researchers and participants were blinded to the participant assignment of the intervention arm. Basic anthropometric measurements (height, weight, gender, and age) were collected at trial initiation. None of the participants used antibiotics or any other probiotics during the study period.

Whole blood, feces, and urine samples were collected from all participants in a hospital at baseline and every 4 weeks thereafter. All samples were labeled with the date of collection and the participant’s identification number. Serum and fecal samples were placed in dry ice during sample transfer to the laboratory. Upon arrival, all samples were immediately stored at −80 °C until subsequent DNA and protein extractions.

### Design of animal experiments

A total of 60 Kunming mice were randomly divided into five groups. The mice were administered with a broad-spectrum antibiotic (Abx) cocktail *via* oral gavage for 10 days to deplete GM^99^. The Cu group received CuSO_4_ at a sublethal concentration of 150 mg/kg body weight (BW)^100^, and the probiotic bacterial strain GR-1 was administered at a dose of 1 × 10^9^ CFU through daily gavage. Fecal samples were regularly collected during the experiment. Blood as well as liver, kidney, and small intestine tissue samples were collected after sacrificing the mice and stored at −80 °C until further analysis. All protocols for animal experiments were approved by the Ethics Committee of Lanzhou University.

To construct the OS model, a total of 70 mice were randomly divided into seven groups. The model mice were exposed to 200 mg/kg bw acetamidophenol (APAP)^57^, 150 mg/kg bw CuSO_4_, and an equal dose of APAP + CuSO_4_ through daily gavage. The intervention groups were orally administered with GR-1 (10^9^ CFU per mouse) or Vc (100 mg/kg bw)^101^ daily to evaluate the antioxidant effect of GR-1. The control and model groups (APAP, Cu, and APAP + Cu) were orally administered with 200 μL skim milk instead. Before sacrifice, fresh fecal samples were collected for anaerobic culture in vitro and a portion of the fecal sample was stored at −80 °C until further use. After sacrifice, liver, kidney, and small intestine tissue as well as venous blood samples were collected and stored at −80 °C.

### Quantification of metal contents

The whole blood, tissue, urine, and fecal samples of human or mice were digested in 65% (v/v) concentrated nitric acid (m(v)/v = 1:5) at 300 °C for 1 h using a digestion furnace (HYP-320; Shanghai Xianjian, China). The levels of Cu and Ni in the samples were determined using a graphite furnace atomic absorption spectrometer equipped with Zeeman background correction (ZEENIT^®^ 700 P, Analytikjena, Germany)^102^ at the Central Laboratory of Lanzhou University. The levels of Cu and Ni in all samples were calculated based on a standard curve^103^.

### Assessment of histopathological parameters

The liver and small intestine tissues of mice were fixed in 4% paraformaldehyde and embedded in paraffin. Subsequently, sections of the liver and small intestine tissues were stained with hematoxylin and eosin^104^ and Alcian blue and periodic acid–Schiff base^105^, respectively.

### Assessment of hematological parameters and biochemical indices

Liver and kidney function tests of all participants were performed in Workers’ Hospital of the Jinchuan Nonferrous Metals Corporation in Jinchang city. The levels of total bilirubin, direct bilirubin, indirect bilirubin, total protein, albumin, globulin, alanine aminotransferase, aspartate aminotransferase, alkaline phosphatase, total bile acid, cholinesterase, urea acid, blood urea nitrogen, and creatinine were detected using the BS2000M automatic biochemical analyzer (Mindray, China). The levels of MDA, CAT, ROS, and total SOD were measured using commercial kits (Nanjing Jiancheng Bioengineering Institute, Nanjing, China). The levels of IL-10, IL-4, IL-1β, and IL-6 (mlbio, ShangHai, China) were measured via enzyme-linked immunosorbent assay according to the manufacturer’s instructions.

### Determination of Cu resistance and antioxidant capacity of probiotics

The probiotic bacteria were cultured overnight in De Man, Rogosa, and Sharpe (MRS) broth at 37 °C with 1% inoculation. After activation, 1 mL of the fermentation broth was inoculated into 100 mL of MRS broth supplemented with different concentrations of CuSO_4_ (0, 10, 15, 20, 30, and 35 mg/L Cu ions) and APAP (0, 10, 20, 30, 40, and 50 mM) and incubated at 37° C under the conditions of stationary culture. After 12 h, the OD_600_ values were measured using a spectrophotometer (METASH V-5100, Shanghai Yuanxi Instrument Co., LTD). The sample was centrifuged at 5,878 ×g for 2 min at 4°C (Beckman Coulter, F2402H), after which the supernatant was collected for determining the 2,2-diphenyl-1-picryl-hydrazyl-hydrate (DPPH) free radical–scavenging ability^2^ and cell pellet was collected for measuring ROS levels (Nanjing Jiancheng Institute of Biotechnology, Nanjing, China).

### Determination of Cu resistance of GM and antioxidant capacity of intestinal contents in mice

To 10 mL of sterile phosphate buffer saline (PBS) (pH = 7.0), 1 g of fresh mouse feces was added. The mixture was blended and then allowed to stand for 2 min at 25 °C to precipitate the large debris. (1) Next, 1 mL of the supernatant was inoculated into 100 mL of sterile basal growth medium^34^ containing 50 mg/kg Cu^2+^ at 37 °C under anaerobic conditions (85% N_2_, 5% O_2_, and 10% CO_2_ in an anaerobic incubator). Sampling was performed every 2 or 3 h, and the OD_600_ value was measured. (2) Then, 1 mL of the supernatant was used to determine the DPPH free radical–scavenging activity^2^ and total antioxidant capacity of intestinal contents (Nanjing Jiancheng Institute of Biotechnology, Nanjing, China). (3) The precipitate of the fecal suspension was resuspended in 5 mL of PBS (pH = 7.0), and cell-free extract was obtained using the ultrasonic cell-break method (Biosafer 650-92)^2^. The sample was centrifuged at 5878 × *g* for 2 min, followed by collection of the supernatant for determining its protein concentration, ROS level, GSH level, and GSH-Px activity (Nanjing Jiancheng Institute of Biotechnology, Nanjing, China).

### Analysis using quantitative RT-PCR

DNA was isolated from feces samples using the TIANamp Stool DNA kit (TIANGEN BIOTECH [BEIJING] CO., LTD.) according to the manufacturer’s instructions. RT-PCR was performed with DNA using strain-specific primers of GR-1^2^ and universal primers of total bacteria^106^ in combination with TB Green^TM^ PCR Premix (TaKaRa, Japan); the reactions were run on the LightCycler 96 RT-PCR system (Applied Biosystems, USA). Before RT-PCR, two different recombinant pMD18T (TaKaRa, Japan) plasmids were prepared and serially diluted to establish the standard curves for GR-1 and total bacteria as described previously^107^. The cycling conditions were as follows: denaturation at 95 °C for 30 s; 50 cycles of extension at 95 °C for 5 s, 60 °C for 30 s, and 95 °C for 10 s; and a final extension at 65 °C–95 °C for 5 s. The absolute copy numbers of GR-1 and total bacteria in the same sample were calculated based on the standard curve. The relative abundance of GR-1 was expressed as follows: copy number of GR-1/copy number of total bacteria × 100.

### Analysis of fecal SCFAs

The concentrations of fecal SCFAs were analyzed via gas chromatography (GC). The samples were prepared as described previously^108^ with minor modification. Briefly, 600 mg feces were homogenized with 1.2 mL of PBS (pH 7.3) and centrifuged at 15,000 × *g* for 15 min at 4 °C. The supernatants were filtered using a 0.22-μm nylon filter (Merck Millipore). An aliquot (800 μL) of the supernatants was acidified by adding 0.4 mL of 50% (v/v) sulfuric acid. After vortexing and standing for 2 min, the organic acids were extracted by adding 0.4 mL of diethyl ether and the supernatants were detected via GC using Trace 1300 (ThermoFisher Scientific, Trace 1300, Singapore) equipped with the Triplus RSH automatic sampler, electron capture detectors, the flame ionization detector capillary columns (TG-WAXMS, 30 m × 0.25 mm inner diameter × 0.25 μm film thickness; Thermo Scientific, USA), and the GC ChemStation software. The injector and detector temperatures were 200 °C and 250 °C, respectively. The column temperature was maintained at 40 °C for 5 min and then increased at rates of 5 °C/min to 150 °C at the first stage, 20 °C/min to 210 °C at the second stage, and 40 °C/min to 300 °C at the third stage. Each SCFA (ethanoic acid, propionic acid, and butyric acid) was quantified based on the standard curve. Total SCFAs were determined as the sum of acetate, propionate, and butyrate; the relative proportion of each SCFA was determined as follows: individual SCFA/total SCFA × 100.

### Sequencing and analysis of 16S rRNA

Fecal microbial DNA from mice and humans was extracted as described above. The integrity of genomic DNA was detected through agarose gel electrophoresis, and the concentration and purity of genomic DNA were determined using the NanoDrop 2000 and Qubit 3.0 spectrophotometers. The V3 and V4 regions of the 16S rRNA gene were first amplified using the primer pairs shown in Supplementary Table 9; then, paired-end sequencing was performed on the Illumina NovaSeq 6000 platform.

#### Illumina read data processing and analysis

Raw reads were processed in the QIIME2 pipeline^109^. Specifically, the “demux” plugin (https://github.com/qiime2/q2-demux) was used for the import of demultiplexed paired-end sequencing reads and creation of the “artifact” file (i.e., qiime2 data format required for subsequent analyses). Furthermore, DADA2^110^ was used to perform quality filtering with --p-max-ee 2, --p-trunk-q 2 command, and chimera filtering (“consensus”). The filtered reads were trimmed using the --p-trim-left-f 23 and --p-trim-left-r 20 commands and truncated using the --p-trunc-len-f 200 and --p-trunc-len-r 200 commands; finally, ASVs were generated^110^. Taxonomic assignments of representative sequences were classified at a confidence threshold of 0.8 against a pretrained naive Bayes classifier, which was trained based on the Ribosomal Database Project database (version 11.5). In this study, samples were rarefied to minimum sample depth (38,691 reads) for subsequent analysis to reduce the bias resulting from different sequencing depths. The α-diversity (observed, Chao1, ACE, Shannon, Simpson, and coverage) of GM among the groups at baseline and study completion was calculated using phyloseq R^111^. PCA was performed to determine the differences in gut microbial communities between the baseline and intervention groups.

### Metagenomic shotgun sequencing and analysis of sample DNA

The total genomic DNA isolated from fecal samples collected at baseline and end of the probiotic administration was mechanically fragmented to an average size of ~400 (200–600) bp using Covaris ME220 (Covaris, USA) for the construction of paired-end libraries according to the manufacturer’s recommended program. Libraries were constructed using the NEBNext® Ultra^TM^ DNA Libraries prep kit for Illumina (NEB, USA). Next, 2 × 150 bp paired-end whole-metagenome sequencing was performed on the Illumina HiSeq 2000 platform using standard protocols at Shanghai GeneSky Biotechnologies Inc.

#### Sequencing data quality control

Raw reads were filtered using the fastp v0.19.5 software^112^ to remove (1) sequences containing >3 N bases, (2) sequences with a Phred score of ≥20 less than 60%, (3) sequences containing resilient sequencing adapters/barcodes, and (4) sequences with a length of <75 bp. Clean reads were subsequently filtered to remove sequences generated from human body using the human genome reference sequence (GRCh38) provided by the University of California, Santa Cruz^113^ using Bowtie 2^114^ with the --very-fast-end-to-end command. The quality of the reads was checked using FastQC v0.11.8^115^.

#### Gene catalog construction and annotation

The clean reads of each sample were assembled using metaSPAdes v.3.15.1^116^ using the --only-assembler -k 31,51,71,91 command, and contigs with a length of ≥500 bp were selected as the assembly result. All redundant contigs were removed using CD-HIT v4.8.1^117^ with parameters “-c 0.95 -aS 0.9 -G 0”. MetaGeneMark v3.38 was used to annotate the assembled contigs^118^. High-quality target genes with a length of ≥100 bp were clustered using CD-HIT v4.8.1^119^ with parameters “-c 0.95 -aS 0.9 -G 0” to obtain a nonredundant set. Binning was performed using metaBAT2 v2.12.1 with default parameters^120^. Based on the National Center for Biotechnology Information nr database, the gene sets for archaea, bacteria, eukaryota, viroids, and viruses were annotated using Diamond (v0.9.36)^121^. The α-diversity, including Shannon and Chao indices, was analyzed and visualized using the vegan package (v2.5.6)^122^ and fossil packages in R (v0.3.7)^123^. DIABLO was used to generate a metagenomic signature using the R package mixOmics v 6.6.2^124^. KEGG annotation was performed using Diamond^121^ against the database with the parameters “bit score ≥60 and e value ≤ 1e−5.” PERMANOVA was performed to analyze between-group differences using the vegan package in R^125^. The differential MGS between the two groups were identified using Wilcoxon rank-sum test^126^ and Metastats^127^. The heatmap of the differential MGS was generated using the OmicShare tool, a free online platform for data analysis (http://www.omicshare.com/tools).

### Metabolomic analysis of serum and fecal samples

Serum and fecal samples stored at −80 °C were thawed on ice. Approximately 50 mg of fecal samples was transferred into 2-mL centrifuge tubes, and 800 μL of 80% methanol was added; afterward, the mixture was ground for 90 s at 70 Hz and vortexed for 30 s using an automatic liquid nitrogen tissue freezing grinder (JXFSTPRP-II-01, Shanghai Jingxin, China). The tubes were ultrasonicated at 4 °C for 30 min. For serum samples, 100 μL of serum was mixed thoroughly with 300 μL of methanol. The mixtures were then ultrasonicated at 4 °C for 30 min, followed by incubation at −40 °C for 1 h, and then vortexed for 30 s. After allowing it to stand for 0.5 h at 4 °C, the solution was centrifuged (10,644 × *g*, 15 min, and 4 °C); the resulting supernatant was transferred into a new centrifuge tube. Next, 5 μL of 0.14 mg/mL DL*-o*-chlorophenylalanine (internal standard) was added into 200 μL of the collected supernatant. After vortexing for 30 s, the mixture was transferred into a bottle for inspection. Untargeted metabolomic analysis was performed via LC–MS (Waters, UPLC; Thermo, Q Exactive). The MS data were acquired in both the positive and negative ion modes. The acquired MS data were processed using the XCMS software. Each ion was identified by combining retention and m/z data. The data annotated the metabolites by matching the exact m/z of samples using the online Human Metabolome and KEGG databases. Features with a variable importance in projection score of ≥1 and Student’s *t* test *p* value of <0.05 were selected. DIABLO was used to evaluate the overall differences in fecal and serum metabolites between the baseline and intervention groups using the R package mixOmics (version 6.6.2).

### Integration of metagenomic and metabolomic data

The analysis of correlation between the metagenomic and fecal or serum metabolomics data was performed using MGS, metagenomic modules, and metabolites that were found to vary significantly between the baseline and intervention groups. Spearman’s correlation analysis was performed using the “corr. test” function in the R package psych (version 1.8.12)^128^. Heatmaps were visualized using the heatmap R package (version 1.0.12).

### Construction of the interaction network of the differential MGS, fecal metabolites, and serum metabolites

The co-occurrence of the differential MGS, fecal metabolites, and serum metabolites was determined based on relative abundances using Spearman’s rank correlation coefficient (*p* < 0.05). The network layout was calculated and visualized using a circular layout via Cytoscape v 3.7.0. All correlations among MGS, fecal metabolites, and serum metabolites were determined.

### Statistical analysis

All data generated in this study were expressed as means ± standard errors of the means. Two-tailed Mann–Whitney test was performed to detect the differences (in HM levels in the blood/feces/urine sample and physiological parameters) between the student and worker groups using GraphPad Prism 8.0.1 for Windows. Kruskal–Wallis test was performed with Dunn’s post hoc analysis to detect differences (in HM levels in the blood/feces/urine sample, SCFA content, and physiological parameters) among more than two groups. An ordinary one-way ANOVA was performed with Tukey’s post hoc analysis to evaluate probiotic colonization at different time points. The association between blood HM levels and age or work duration was investigated using two-tailed Spearman’s correlation analysis, which was performed using SPASS v21 and plotted using OriginPro 8.0. Statistical significance was set at *p* < 0.05.

For 16S rRNA gene sequencing, the α-diversity estimates (observed, Chao1, ACE, Shannon, Simpson, and coverage) among the six groups (LC, MC, HC, PTLC, PTMC, and PTHC) were calculated using Kruskal–Wallis test. PCA plots were generated based on the Bray–Curtis distance of ASVs.

For metagenomics, the α-diversity estimates (Chao, Shannon, and InvSimpson) between baseline and intervention groups were calculated using Wilcoxon rank-sum tests (Fig. 3a). Both Wilcoxon rank-sum test and Metastats were used to detect differences in GM (relative abundance of species) between the two treatment groups. DIABLO from the R package mixOmics v6.6.2^124^ was used to differentiate between MGS of the baseline and intervention groups (Fig. 3b). GM signatures between the two groups (baseline- or intervention-enriched) were identified using Metastats (Fig. 3c). The MGS that showed significant differences in abundance between the baseline and intervention groups were evaluated using Wilcoxon rank-sum test (Fig. 3d). Differentially enriched KEGG modules or pathways were evaluated using Wilcoxon rank-sum test after Benjamini–Hochberg adjustment.

Regarding the metabolome, DIABLO from the R package mixOmics v6.6.2^124^ was used to differentiate between participants from the baseline and intervention groups (Fig. 4a, b). Procrustes analysis of fecal metabolome versus serum metabolome was performed using the R *vegan* package, and the Procrustes *p* value was generated based on 999 permutations^55^ (Fig. 4c). The differential fecal and serum metabolites between the two groups were identified using Student’s *t* test (Fig. 4d, e).

Power analyses were performed using the method developed by Falony et al.^129^ (Fig. 5a). Correlations between the relative abundance of MGS and fecal/serum metabolites involved in antioxidation or OS were evaluated using Spearman’s correlation analysis (Fig. 5b). The differential abundances of serum and fecal metabolites associated with OS and antioxidation were evaluated using Student’s *t* test. Differentially enriched KEGG modules were identified using Wilcoxon rank-sum test (Fig. 5c).

## Supplementary information

Supplementary information

## Data Availability

Sequence data of the 16S rRNA and metagenome have been deposited in the National Center for Biotechnology Information under accession numbers PRJNA753301 and PRJNA753698, respectively. The raw data of serum and fecal metabolomics are available on the MetaboLights database under the accession number MTBLS3434. The source data underlying Figs. 2–8 and Supplementary Figs. 2–8 are provided as a source data file. The full trial protocol and data generated in this study are available from the corresponding authors upon request.

## Source data

source data
