# Supplementary material for: Human supplementation with Pediococcus acidilactici GR-1 decreases heavy metals levels through modifying the gut microbiota and metabolome
Source: NPJ Biofilms Microbiomes. 2022 Aug 16;8:63. doi: 10.1038/s41522-022-00326-8 (PMC9381558; doi:10.1038/s41522-022-00326-8)
Supplement: Supplementary file 1 — Supplementary information [file 41522_2022_326_MOESM1_ESM.pdf]

SUPPLEMENTARY INFORMATION

Title

Human supplementation with *Pediococcus acidilactici* GR-1 decreases heavy metals levels through modifying the gut microbiota and metabolome

Authors

Pengya Feng [a,#], Jinfeng Yang [a,#], Shuai Zhao [a,#], Zhenmin Ling [a], Rong Han [b], Ying Wu [a], EI-Sayed Salama [c], Apurva Kakade [a], Aman Khan [a], Wei lin Jin [d], Weibing Zhang [b], Byong-Hun Jeon [e], Jingjing Fan [a], Minrui Liu [b], Tursunay mamtimin [a], Pu Liu [a, *], Xiangkai Li [a, *]

a. Ministry of Education Key Laboratory of Cell Activities and Stress Adaptations, School of Life Science, Lanzhou University, Lanzhou, Gansu 730000, P. R. China
b. College of Life Science and Technology, Gansu Agricultural University, Lanzhou, 730070, PR China
c. Department of Occupational and Environmental Health, School of Public Health, Lanzhou University, Lanzhou, Gansu 730000, P. R. China
d. Institute of Cancer Neuroscience, Medical Frontier Innovation Research Center, The first Hospital of Lanzhou University, The First Clinical Medical College of Lanzhou University, Lanzhou 730000, P. R. China
e. Department of Earth Resources and Environmental Engineering, Hanyang University, Seoul 04763, South Korea

*Correspondence: xkli@lzu.edu.cn; liupu@lzu.edu.cn.Tel.: +86-931-8912560

# These authors contribute equally to this work.

TABLE OF CONTENTS

SUPPLEMENTARY TABLE P 3-23

SUPPLEMENTARY FIGURE P 24-36

SUPPLEMENTARY TABLE

Supplementary Table 1 Blood and urine levels of Cu and Ni at the time of recruitment and comparisons to the levels found in a developed country.

**Study group**	**Sample category**	**Students (H)** AVE±SD (Range)	**Occupational workers (O)** AVE±SD (Range)	**FC (O/H)**	**FC (H/RV [a])**	**FC (O/RV [a])**
**Cu**	**Blood**	627.7±106.7 (470.6-938.6)	1260.94±213.7 (851.0-2205)	2.00	0.63	1.26
	**Urine**	28.30±14.54 (7.433-55.5)	53.64±32.79 (20.43-337.4)	1.90	1.13	2.15
**Ni**	**Blood**	1.100±0.3020 (0.6829-1.936)	4.960±3.323 (0.02281-13.10)	4.51	1.00	4.51
	**Urine**	3.963±1.983 (1.204-8.97)	16.19±10.82 (5.308-70.63)	4.09	0.90	1.41

H, students as healthy control; **O**, occupational workers; **RV**, the reference value; **FC**, fold change.

a. The RV of blood Cu and Ni level is 1000 ug· L^{-1} (1000-1100 ug· L^{-1}) for males and 1.1 ug· L^{-1} (0.85-1.3 ug· L^{-1}), respectively, and the RV of urine Cu and Ni level is 25 ug·L^{-1} (22-28 ug·L^{-1}) and 4.4 ug· L^{-1} (3.8-5.0 ug·L^{-1}), respectively, which are based on the Canadian health measures survey 2007-2013 (Saravanabhavan, Werry et al. 2017).

Supplementary Table 2 The composition and dose of the yogurt used in this study.

	**Probiotic composition**	**Dosage**	**Duration**
Probiotic yogurt	*Lactobacillus bulgaricus* (commercial)<br>*Streptococcus thermophilus* (commercial)<br>*Pediococcus acidilactici* GR-1 (laboratory)	10^{10} cfu of each bacteria in one carton of probiotic yogurt （250g） consumed daily	12 weeks
Conventional yogurt	*Lactobacillus bulgaricus* (commercial)<br>*Streptococcus thermophilus* (commercial)	10^{10} cfu of each bacteria in one carton of conventional yogurt (250g) consumed daily	12 weeks

Supplementary Table 3 Blood heavy metals level and biochemical measurements at baseline in worker participants.

Characteristics	Baseline	Baseline	*P* value
	**Conventional yogurt (n=76)**	**Probiotic yogurt (n=76)**	
Blood Cu (μg/L)	1275±219.9	1246±207.7	0.4171
Urine Cu (μg/L)	55.63±38.81	55.64±25.67	0.9979
Blood Ni (μg/L)	5.065±3.271	4.855±3.392	0.6982
Urine Ni (μg/L)	16.18±10.76	16.20±10.95	0.9934
TBIL(μmol/L)	15.63±8.478	15.02±6.082	0.6140
DBIL(μmol/L)	5.593±2.948	5.353±2.133	0.5659
IBIL(μmol/L)	10.04±5.614	9.605±4.154	0.5893
TP (g/L)	77.05±3.813	78.05±3.303	0.0844
ALB (g/L)	45.99±2.122	46.54±1.763	0.0880
GLB (g/L)	31.05±2.998	31.52±2.833	0.3306
A/G	1.494±0.1508	1.488±0.1444	0.7882
PA (mg/dL)	26.33±4.658	27.55±4.119	0.1508
ALT (U/L)	30.53±19.50	26.21±13.88	0.1180
AST (U/L)	23.99±8.255	23.01±5.821	0.4020
AST/ALT	1.218±0.4153	1.094±0.378	0.3696
ALP (U/L)	97.50±25.46	93.30±24.27	0.2999
GGT (U/L)	40.71±47.05	35.58±25.85	0.4060
TBA (μmol/L)	5.726±4.861	5.849±3.822	0.8628
CHE (U/L)	8691±1937	9074±1598	0.1862
BUN (mmol/L)	5.684±1.229	5.558±1.10	0.5084
UA (μmol/L)	344.4±88.73	334.4±78.89	0.4645
CREA (μmol/L)	70.75±10.35	72.14±10.17	0.8128

All data are expressed as mean ± standard deviation (SD). Analysis of two-tailed Mann-Whitney U was performed to compare the two groups at baseline. Data with p>0.05 are

not significant. TBIL, total bilirubin; DBIL, direct bilirubin; IBIL, indirect bilirubin; TP, total protein; ALB, albumin; GLB, globulin; A/G, the ratio of albumin to globulin; PA, prealbumin; ALT, alanine aminotransferase; AST, aspartate amino transferase; ALP, alkaline phosphatase; GGT, γ-gultamyltransferase; TBA, total bile acid; CHE, cholinesterase; BUN, urea nitrogen; UA, uric acid; CREA, creatinine. The reference value for TBIL, DBIL, IBIL, TP, ALB, GLB, A/G, PA, ALT, AST, ALP, TBA, CHE, BUN was 2.0-20.4, 0.0-7.0, 3.00-15.00, 62.0-85.0, 35.0-53.0, 20.00-35.00, 1.50-2.50, 10.00-40.00, 0-38, 0-38, 0.00-3.00, 40-150, 0.00-15.00, 4000-11000, 1.7-8.3, respectively. Data were presented as mean ± SD.

Supplementary Table 4 Interval division based on reference value from Canadian health measures survey 2007-2013 (Saravanabhavan, Werry et al. 2017).

Ni (μg/L)	Cu (μg/L)	Level grade
1.1	1000	Normal value
<1.1	<1000	Low
1.1-6.0	1000-1300	Medium
>6.0	>1300	High

Supplementary Table 5 Effect of 12-week intervention with conventional yogurt and probiotic yogurt on hepatic and renal function characteristics in the whole group of participants.

**Characteristics**	**Baseline Conventional yogurt**	**Intervention Probiotic yogurt**	**Baseline Conventional yogurt**	**Intervention Probiotic yogurt**	***P* for Conventional yogurt**	***P* for Probiotic yogurt**
TBIL(μmol/L)	15.55±8.313	15.68±5.14	14.96±6.019	12.89±6.273	0.9419	0.1095
DBIL(μmol/L)	5.526±2.904	5.371±2.05	5.336±2.109	4.667±2.113	0.7986	0.1128
IBIL(μmol/L)	10.03±5.5	10.31±3.285	9.557±4.111	8.224±4.34	0.8026	0.1431
TP (g/L)	77.24±3.829	74.74±3.073	77.94±3.337	73±3.415	0.0027	<0.0001
ALB (g/L)	45.97±2.084	45.24±1.959	46.48±1.794	43.83±1.823	0.1101	<0.0001
GLB (g/L)	31.26±3.097	29.5±2.391	31.46±2.831	29.17±3.026	0.0081	0.0002
A/G	1.485±0.1547	1.543±0.1393	1.488±0.1444	1.518±0.1758	0.0868	0.2802
PA (mg/dL)	26.19±4.700	33.28±4.998	27.44±4.201	24.97±5.500	<0.0001	0.0178
ALT (U/L)	30.09±19.17	33.3±16.05	26.69±14.46	26.24±15.57	0.4362	0.8808
AST (U/L)	23.95±8.080	24.19±6.221	23.12±5.889	22.85±8.247	0.8906	0.8488
AST/ALT	1.203±0.4122	1.338±0.4413	1.106±0.378	1.161±0.5276	0.1501	0.9004
ALP (U/L)	96.89±25.86	92.56±28.11	93.15±24.13	88.43±21.01	0.4632	0.2314
GGT (U/L)	39.48±46.17	34.3±15.58	35.53±25.64	39.29±77.33	0.5696	0.0533
TBA (μmol/L)	5.581±4.782	3.944±2.185	5.767±3.807	6.080±3.208	0.0893	0.9539
CHE (U/L)	8611±1925	5609±2145	9061±1580	8618±1501	<0.0001	0.1911

BUN (mmol/L)	5.605±1.261	5.343±1.215	5.515±1.12	5.108±1.395	0.3486	0.1212
UA (μmol/L)	339.6±89.46	346.3.±58.25	333.5±78.32	347.1±90.11	0.7192	0.6431
CREA (μmol/L)	70.73±11.06	79.19±15.02	72.24±10.12	67.97±13.08	0.0023	0.0529

All data are expressed as mean ± standard deviation (SD). Analysis of two-tailed Mann-Whitney U was performed to compare the change in each biochemical measure at baseline and intervention groups. Data with $p<0.05$ are significant. TBIL, total bilirubin; DBIL, direct bilirubin; IBIL, indirect bilirubin; TP, total protein; ALB, albumin; GLB, globulin; A/G, the ratio of albumin to globulin; PA, prealbumin; ALT, alanine aminotransferase; AST, aspartate amino transferase; ALP, alkaline phosphatase; GGT, γ-gultamyltransferase; TBA, total bile acid; CHE, cholinesterase; BUN, urea nitrogen; UA, uric acid; CREA, creatinine. The reference value for TBIL, DBIL, IBIL, TP, ALB, GLB, A/G, PA, ALT, AST, ALP, TBA, CHE, BUN was 2.0-20.4, 0.0-7.0, 3.00-15.00, 62.0-85.0, 35.0-53.0, 20.00-35.00, 1.50-2.50, 10.00-40.00, 0-38, 0-38, 0.00-3.00, 40-150, 0.00-15.00, 4000-11000, 1.7-8.3, respectively.

Supplementary Table 6 The distribution of the 6 random selected samples at different gradients.

Category	Grade	Sample number					
		1	2	3	4	5	6
Blood Ni	Low level		A80				
	Medium level	A77				A130	A151
	High level			A91	A119		
Blood Cu	Low level	A77					
	Medium level		A80			A130	
	High level			A91	A119		A151

Supplementary Table 7 The basic characteristics of the 6 random selected samples.

Sample Number	Heavy metals in blood (μg/L)		Age (years)	Working time (years)
	Ni	Cu		
A77	3.245	989.167	43	25
A80	0.700	1047.321	34	12
A91	9.023	1350.667	33	12
A119	6.957	1504.500	46	25
A130	2.533	1102.500	44	25
A151	2.823	1614.500	53	28

Supplementary Table 8 Oxidative stress defense relevant KEGG modules in faeces of workers before and after 12 weeks of probiotic yogurt intervention. KEGG modules that significantly differed in abundance between the two groups were calculated using Wilcoxon rank-sum test after Benjamini-Hochberg adjustment.

ID	Description	Gene	Pathways	Mean_baseline	Mean_intervention	p.value	p.adjust(BH)
K03781	catalase [EC:1.11.1.6]	katE, CAT, catB, srpA	ko00380 Tryptophan metabolism; ko00630 Glyoxylate and dicarboxylate metabolism; ko01100 Metabolic pathways; ko01110 Biosynthesis of secondary metabolites; ko01130 Biosynthesis of antibiotics; ko01200 Carbon metabolism; ko04011 MAPK signaling pathway-yeast; ko04016 MAPK signaling pathway-plant; ko04068 FoxO signaling pathway; ko04146 Peroxisome;ko04211 Longevity regulating pathway; ko04212 Longevity regulating pathway-worm; ko04213 Longevity regulating pathway-multiple species; ko05014 Amyotrophic lateral sclerosis (ALS)	42.22119167	48.18196017	0.588745	0.806805
K00432	glutathione peroxidase [EC:1.11.1.9]	gpx, btuE, bsaA	ko00480 Glutathione metabolism; ko00590 Arachidonic acid metabolism; ko01100 Metabolic pathways; ko04918 Thyroid hormone synthesis	47.438582	70.37770867	0.309524	0.686271

K00383	glutathione reductase (NADPH) [EC:1.8.1.7]	GSR, gor	ko00480 Glutathione metabolism; ko01100 Metabolic pathways; ko04918 Thyroid hormone synthesis	68.15360117	111.1892782	0.24026	0.625488
K16015	oxidoreductase [EC:1.1.1.-]	rifL, asm44	ko01051 Biosynthesis of ansamycins; ko01130 Biosynthesis of antibiotics	0.021367333	0.407070167	0.599266	0.815575
K04565	superoxide dismutase, Cu-Zn family [EC:1.15.1.1]	SOD1	ko04146 Peroxisome; ko04213 Longevity regulating pathway - multiple species; ko05014 Amyotrophic lateral sclerosis (ALS); ko05016 Huntington disease; ko05020 Prion diseases	4.2935735	14.54515067	0.041126	0.389241
K04564	superoxide dismutase, Fe-Mn family [EC:1.15.1.1]	SOD2	ko04013 MAPK signaling pathway-fly; ko04068 FoxO signaling pathway; ko04146 Peroxisome; ko04211 Longevity regulating pathway; ko04212 Longevity regulating pathway worm; ko04213 Longevity regulating pathway-multiple species	65.32628383	110.2628747	0.041126	0.389241

Supplementary Table 9 Differential metabolites in feces of workers at baseline and after 12 weeks of probiotic yogurt intervention. The differential faecal metabolites between the two groups were identified using Student's t-test.

ID	MS2 name	Mean_baseline	Mean_intervention	VIP	p-value	q-value	Log_FC
35	Piperidine	0.00089187	2.9255E-05	1.94648966	0.01107365	0.13874672	4.930073107
142	4-aminobutyric acid	0.00026311	7.813E-05	1.46156385	0.03354137	0.1620275	1.751731891
144	Y-Aminobutyric acid (GABA)	0.00250005	0.0007187	1.52330548	0.02906448	0.15892217	1.798496949
152	Choline	0.000418	0.00015832	1.7520613	0.00233431	0.10345109	1.400660522
290	Taurine	0.00052744	3.3149E-05	1.96384812	0.00040262	0.05196703	3.991974434
355	Creatine	0.00652739	0.00055076	1.67652616	0.03649236	0.16393824	3.567019069
726	N-Acetyl-L-aspartic acid	0.00020073	7.9557E-05	1.47967159	0.01036731	0.13760357	1.33518674
727	N-Acetyl-DL-aspartic acid	0.00023305	9.3598E-05	1.42186754	0.04599342	0.17084305	1.316056461
834	N-(5-acetamidopentyl)acetamide	0.00077649	3.1682E-05	1.89065562	0.03173694	0.16075089	4.61522961
969	N3,N4-Dimethyl-L-arginine	0.00014307	7.4381E-05	1.38523103	0.0140999	0.1433718	0.943771345
1131	N-Acetyl-α-D-glucosamine	0.00269578	0.00083821	1.67429708	0.01581976	0.14533962	1.685315784
1374	2'-Deoxyadenosine	9.4754E-05	3.5368E-05	1.61176011	0.03443687	0.16263706	1.421729242
1531	Apigenin	3.5333E-05	1.8514E-06	1.82599217	0.01697114	0.14645767	4.254284094
1708	Catechin	2.7801E-05	1.1086E-06	1.95356168	0.04868829	0.17253734	4.648343176
1807	Sphingosine (d18:1)	0.00023045	5.7003E-05	1.38038774	0.04698302	0.17148387	2.015323079
1920	Heptadecanoyl Ethanolamide	7.1866E-05	1.1457E-05	1.43521749	0.030785	0.16013122	2.649119878

1954	2-Amino-1,3,4-octadecanetriol	5.7294E-05	3.6102E-05	1.60375862	0.00458345	0.11831785	0.666289777
1985	alpha-Linolenoyl Ethanolamide	0.0002876	6.4197E-05	1.53933949	0.02867503	0.15863111	2.163510702
1998	Linoleoyl Ethanolamide	0.00762935	0.00190668	1.55095276	0.01670286	0.14620946	2.000495422
2350	3α-Hydroxy-12 Ketolithocholic Acid	0.0003272	7.5842E-05	1.57439561	0.0382835	0.1649728	2.109114826
2393	Palmitoylcarnitine	0.00034962	0.00016058	1.52440275	0.01692495	0.14641543	1.122461728
2549	Andrastin D	0.00028161	1.1348E-05	1.90421231	0.02448163	0.15500221	4.633172337
2769	PC (16:0/0:0)	0.00172581	0.00042462	1.74100764	0.0134879	0.14256618	2.023015002
2910	Bilirubin	0.00011606	1.7011E-05	1.65909391	0.01693569	0.14642527	2.770379804
202	citramalate	0.00025526	5.5939E-05	1.84854269	0.00840446	0.08372178	2.190044666
217	Ribose	0.00164088	0.00072447	1.7722071	0.00021327	0.02456745	1.179482146
247	Gentisic acid	0.00020877	6.9034E-05	1.41806225	0.01341094	0.0950455	1.596501965
308	3-Hydroxy-3-methylglutaric acid	0.00049954	5.7374E-05	1.62178402	0.02891579	0.11429537	3.122140237
323	1,5-Anhydro-D-glucitol	0.00027658	0.00011372	1.48273768	0.00866801	0.08437272	1.282147883
341	3-Phenyllactic acid	2.3133E-05	4.9222E-06	1.6275785	0.04462692	0.12287851	2.232577376
456	Glucose	0.0135564	0.00584183	1.32965171	0.00908524	0.08547656	1.214482374
572	1H-2-benzopyran-1-one, 6,8-dihydroxy-3-methyl-	1.7963E-05	1.2944E-06	1.59211782	0.02697198	0.11268411	3.794622669
579	D-Glucuronic Acid	0.00325739	0.0010311	1.49174958	0.01741064	0.10107813	1.659531928
853	Ethyl-beta-glucuronide	0.00063929	0.00038225	1.36829588	0.02351766	0.1093169	0.74196871
873	Sinapinic acid	1.7937E-05	3.5441E-06	1.45679391	0.0493069	0.12451191	2.339398857
1156	N-Acetyltryptophan	5.2171E-05	1.4879E-05	1.27422518	0.0255006	0.11133693	1.809920947

1428	Inosine	7.7257E-05	3.5386E-05	1.27713744	0.03534798	0.11857644	1.12650434
1452	Genistein	0.00022442	1.5543E-05	1.72456428	0.02796978	0.11353348	3.851883201
1523	Phloretin	3.7151E-05	7.6079E-06	1.29721216	0.02171855	0.10724409	2.287848386
1712	Catechin	0.0001354	3.9583E-05	1.14035913	0.0107378	0.08949917	1.774272363
1921	Arachidonic acid	0.00335861	0.00196016	1.12319766	0.04389423	0.12259567	0.776889839
1932	Epigallocatechin	8.6871E-05	1.0223E-05	1.50345137	0.01450798	0.09689427	3.087027089
1974	N-Acetylneuraminic acid	0.00146891	0.00053814	1.3482636	0.00952099	0.08663452	1.448695892
2090	zearalenone	4.5058E-05	1.6487E-05	1.0763694	0.03910731	0.12052735	1.450476985
2134	20-Hydroxy-(5Z,8Z,11Z,14Z)-eicosatetraenoic acid	9.6319E-05	6.5077E-06	1.71576807	0.03186658	0.11643453	3.887603857
2217	3-Hydroxy-3',4',5'-trimethoxyflavone	4.9703E-05	3.115E-06	1.6714844	0.04191748	0.12179047	3.996019597
2737	Testosterone sulfate	0.00103402	0.0004353	1.00928958	0.01438044	0.09669036	1.248177687
3054	Deoxycholic Acid	0.01949122	0.00078415	1.75337112	0.0367521	0.1193442	4.635545937
3203	3-Dehydrocholic acid	0.00018367	2.3906E-05	1.63898941	0.01433192	0.09661207	2.941638382
3227	Cholic acid	0.03312883	0.00101466	1.82218202	0.02507126	0.11092064	5.029020071
3228	Cholic acid	0.00553497	0.00045292	1.7372343	0.00753523	0.08134087	3.611259203
3234	α-Muricholic Acid	6.7462E-05	2.9699E-05	1.452389	0.02045938	0.10563269	1.183680911
3373	Otenzepad	1.663E-05	3.365E-07	1.87512401	0.02768331	0.11329461	5.627063196
3478	Vitexin	2.8047E-05	1.1138E-06	1.86190316	0.04931418	0.12451424	4.654296477
3782	Andrastin C	3.1654E-05	1.1972E-05	1.22514736	0.03050884	0.11549188	1.402758978
4454	Cochlioquinone A	5.9915E-05	1.9795E-05	1.30462356	0.02546592	0.11130371	1.597806238

4762	Biliverdin	3.7422E-05	1.4939E-05	1.79834827	0.00510119	0.0726009	1.324799647
4772	Bilirubin	0.0002143	3.4917E-05	1.44182075	0.04241427	0.12199888	2.61759665
5129	L-alpha-Phosphatidylethanolamine (Soy)	0.00085761	0.00027474	1.42710889	0.03594611	0.1189096	1.642246641
2292	(-)-Riboflavin	2.6029E-05	4.0565E-05	1.39025869	0.02637849	0.15676565	-0.64011479
1853	(R)-2-hydroxystearic acid	0.01503862	0.04909464	1.74704473	0.0304665	0.11546138	-1.70689358
542	10-Hydroxydecanoic acid	1.0001E-05	3.0089E-05	1.41097629	0.02248512	0.10815804	-1.58911751
1843	13,14-Dihydro-15-keto-tetranor prostaglandin F1α	3.2268E-06	4.9868E-05	1.92207103	0.00584577	0.07535281	-3.94995762
2810	19(R)-hydroxy Prostaglandin F1α	1.3317E-05	2.5406E-05	1.23884224	0.04683077	0.12368297	-0.93197962
1297	2',4',6'-Trihydroxydihydrochalcone	2.7946E-07	5.389E-05	1.48247535	0.04606057	0.1234094	-7.59124145
649	4-Phenylbutyric acid	1.2749E-05	4.0928E-05	1.55454645	0.03602902	0.16365614	-1.68269368
893	5-Hydroxyindole-3-acetic acid	8.2796E-05	0.00068956	1.50668259	0.04276858	0.16858777	-3.05804147
470	8-Hydroxyquinoline	1.0224E-05	0.00010488	1.52722621	0.04274269	0.16856855	-3.35870729
1128	beta-Hydroxymyristic acid	2.2416E-05	6.7562E-05	1.42597656	0.04242033	0.12200139	-1.5917066
414	Citrulline	0.00024806	0.00049895	1.44087786	0.01572795	0.09871502	-1.00821627
228	D-(+)-Proline	0.00152817	0.00404606	1.67847936	0.02087736	0.15092625	-1.40471169
321	D-(+)-Pyroglutamic Acid	0.00127907	0.00238483	1.50832657	0.00629074	0.12697506	-0.89879091
2462	delta4-Dafachronic acid	5.1637E-06	5.0063E-05	1.72859262	0.04262688	0.16848233	-3.27727971
819	Ecgonine	5.3314E-05	0.00011239	1.58375458	0.00774301	0.13183213	-1.07591538
1808	Enterolactone	0.00034342	0.0032169	1.52884261	0.02953879	0.11477566	-3.22763657
1284	Ethyl myristate	0.00369079	0.00632754	1.14019727	0.03489347	0.11831694	-0.77771608

719	Gly-Val	0.00012701	0.00020458	1.48140105	0.04318714	0.16889596	-0.68777792
237	Indole	2.1896E-05	0.00010715	1.55855733	0.02432056	0.15484179	-2.29095528
511	L-(-)-Methionine	0.00096289	0.00171406	1.6030135	0.04282041	0.1686262	-0.83198131
328	L(-)-Pipecolinic acid	0.00012387	0.00074228	1.83802615	0.03053059	0.15995992	-2.58320058
732	L-(+)-Citrulline	0.00078326	0.00153464	1.75832511	0.00309746	0.11041711	-0.9703423
364	L(+)-Ornithine	0.00149864	0.00387479	1.57018906	0.02388088	0.15439464	-1.37046342
693	Levetiracetam	7.9385E-05	0.00012817	1.60120759	0.0107436	0.13822893	-0.69111283
494	L-Glutamic acid	0.00441469	0.01051381	1.36747773	0.01262982	0.14132317	-1.25190058
358	L-Isoleucine	0.0036735	0.00781582	1.84773736	0.00885648	0.13463682	-1.08924182
2859	Lithocholic Acid	0.01125048	0.03587107	1.54542624	0.00187539	0.05244701	-1.67283413
357	L-Norleucine	0.00985051	0.01799182	1.70884612	0.02409493	0.15461404	-0.86907041
779	L-Tyrosine	0.00170198	0.00285989	1.62816913	0.02251635	0.15291436	-0.74874269
2722	Nervonic acid	0.00012499	0.00032457	1.15128395	0.03815016	0.12006136	-1.37668479
272	N-Tigloylglycine	1.0862E-05	6.5009E-05	1.43796044	0.04736253	0.12386734	-2.58133266
1607	Oleic acid	0.01068824	0.03338731	1.72839907	0.01914973	0.10379306	-1.64327606
532	Oxypurinol	0.00011384	0.00019345	1.23599703	0.04005175	0.16646185	-0.76503463
2847	Riboflavin	5.6448E-05	8.4911E-05	1.33542408	0.04795604	0.12406891	-0.58901458
299	Thymine	4.7804E-05	0.00039556	1.87836296	0.02566316	0.15612665	-3.04870133
1442	trans-10-Heptadecenoic Acid	0.00046343	0.00128682	1.17086585	0.0352624	0.118528	-1.47339344
202	Uracil	0.00010901	0.00022965	1.62698919	0.00142261	0.08895675	-1.07503799

421	Urocanic acid	7.1287E-05	0.00022085	1.77706763	0.02716849	0.15743785	-1.63133182
530	Xanthine	0.0003712	0.00061903	1.25686694	0.03094868	0.16024014	-0.73779387
1572	α-Linolenic Acid	0.00079199	0.00260225	1.49376968	0.00434549	0.06944626	-1.71621557

Supplementary Table 10 Differential metabolites in serum of workers at baseline and after 12 weeks of probiotic yogurt intervention. The differential serum metabolites between the two groups were identified using Student's t-test

ID	MS2 name	Mean_baseline	Mean_intervention	VIP	p-value	q-value	Log_FC
920	(+/-)13-HODE	0.00011502	5.8799E-05	1.52059933	0.02323464	0.06020311	0.9679836
1086	(±)11(12)-EET	0.00173107	0.00037872	1.95401506	7.6335E-05	0.00248948	2.19247814
963	(R)-2-hydroxystearic acid	0.00054034	0.00028179	1.9762567	3.4757E-06	0.00073171	0.9392459
1005	11(Z),14(Z)-Eicosadienoic acid	0.00069242	0.00025529	1.86000562	2.0893E-05	0.00135803	1.43949893
791	16-Hydroxyhexadecanoic acid	6.3768E-05	4.0405E-05	1.6046485	0.00328854	0.01872285	0.65830381
1121	2-Amino-1,3,4-octadecanetriol	0.00112091	0.00028864	1.64511831	0.03527412	0.09933197	1.95734901
346	2-Hydroxyhippuric acid	0.00011771	2.8676E-05	1.72867913	0.03153293	0.07177659	2.03729534
998	8Z,11Z,14Z-Eicosatrienoic acid	0.00124741	0.00038618	1.9297752	9.6E-06	0.00102351	1.69156871
1037	Arachidic Acid	7.8773E-05	6.0422E-05	1.379748	0.01970766	0.054439	0.38262016
985	Arachidonic acid	0.01136322	0.0059108	1.70075433	0.00176198	0.01381423	0.94294665
1586	Calcitriol	3.7702E-05	2.5882E-05	1.37697328	0.01877567	0.0530823	0.54268868
106	Choline	0.00997761	0.00620133	1.89911289	9.8694E-05	0.00234909	0.6861162
977	cis-5,8,11,14,17-Eicosapentaenoic acid	0.00108926	0.00041244	1.39667154	0.01815569	0.05214394	1.40110483
490	Cotinine	0.00035184	4.8957E-06	1.87648653	0.0261385	0.08447696	6.16724402
207	D-(+)-Pipecolinic acid	0.00053953	0.00031536	1.36820104	0.01596321	0.06360348	0.77470282
1457	Deoxycholic Acid	0.00058905	0.00029937	1.14001042	0.03970194	0.08184039	0.97648851

1247	D-Erythro-sphingosine 1-phosphate	0.00021066	0.00015786	1.74908801	0.00128269	0.01216007	0.41626772
1068	Dihydrosphingosine	0.00064412	0.0005095	1.70909798	0.00230684	0.0172767	0.33823038
153	DL-Mandelic acid	2.4497E-05	1.3496E-05	1.40756364	0.01239257	0.04174985	0.86007222
1168	Docosatetraenoic acid	0.00085565	0.00036519	1.70386393	0.00041967	0.00636068	1.22836899
697	Ethyl myristate	0.00134033	0.00069617	1.74399113	0.00122242	0.01117105	0.9450632
159	Gentisic acid	3.4077E-05	1.1388E-05	1.85253232	0.00798071	0.03137194	1.58129659
129	Glutamic acid	0.00056779	0.00034107	1.64725352	0.00191954	0.01445007	0.73527716
163	Histidine	0.00067677	0.00037899	1.36504744	0.01619266	0.04896605	0.8364983
221	Homogentisate	0.00048767	0.0001645	1.45397265	0.0102088	0.03680575	1.56778719
232	L(+)-Ornithine	0.00015597	0.00011047	1.51478131	0.00699685	0.03621479	0.49755192
347	L-Glutamic acid	0.00092138	0.00047402	1.95370389	3.621E-05	0.00110281	0.95885973
393	L-Histidine	0.00075577	2.6615E-05	1.94639589	0.02700777	0.08594741	4.82764154
835	Linoleic acid	0.02791307	0.01849807	1.38712343	0.04468776	0.08713187	0.59356574
204	L-Pyroglutamic acid	0.00082986	0.00057995	1.86499102	0.00022752	0.00377143	0.51694129
528	Myristic Acid	0.00014014	7.478E-05	1.74766909	0.00208519	0.01506614	0.90610846
782	N,N-Dimethyldodecylamine N-oxide	0.00016093	9.3739E-05	1.38497145	0.02980321	0.09081906	0.77973181
455	Nigrosporapyrone D	0.00050948	0.00014209	1.61552235	0.01563089	0.06281171	1.84226628
64	N-Methyl-2-pyrrolidone	0.08278719	0.07260576	1.41841118	0.02018403	0.07302546	0.18932354
850	Oleic acid	0.0312798	0.01793559	1.63251228	0.00373234	0.02019379	0.80240609
1142	Oleoyl ethanolamide	2.1464E-05	1.324E-05	1.53379114	0.01923854	0.07095054	0.69704921

683	Palmitoleic acid	0.00138851	0.00059557	1.53503723	0.02635445	0.06491127	1.22119518
725	Pantothenic acid	3.1468E-05	1.6965E-05	1.61011523	0.01756801	0.06724556	0.89134779
1426	PC(16:0/0:0)	0.01011811	0.00668786	1.81751731	0.0002372	0.00384419	0.59732469
1752	Phosphatidylethanolamine lyso 16:0	0.00114236	0.00061535	1.65487951	0.01305236	0.04311858	0.89253101
1902	Phosphatidylethanolamine lyso 18:0	0.00161041	0.00106501	1.5463469	0.0055344	0.02528773	0.59655418
1997	Phosphatidylethanolamine lyso 20:4	0.00016707	0.00013391	1.48689152	0.0100455	0.03640803	0.31917154
529	Phosphocholine	3.9002E-05	2.4113E-05	1.2954403	0.0370302	0.10180468	0.6937525
1055	Sphingosine (d18:1)	0.00013536	4.1747E-05	2.06762792	3.3282E-05	0.00104255	1.69705462
1353	Thromboxane B2	5.9213E-05	4.8785E-06	2.00729714	0.00062855	0.00765856	3.60140309
454	Uric acid	0.00104459	0.00080253	1.23081307	0.03699351	0.1017542	0.38032192
1310	Ursocholanic Acid	2.8699E-05	1.3746E-05	1.75721954	0.00098013	0.00997261	1.06198852
824	α-Linolenic Acid	0.00413907	0.00201419	1.50019059	0.00865415	0.03303686	1.03910492
100	N,N-Dimethylglycine	0.00019811	0.00031376	1.71588783	0.00344424	0.02255168	-0.66334797
103	Choline	0.00019818	0.00075198	2.02218993	1.1898E-06	0.00050698	-1.92389829
168	L-Valine	0.00323546	0.0042786	1.27803451	0.04074843	0.10685293	-0.40316545
176	Threonine	0.0004975	0.0007453	1.50902741	0.03009927	0.09131537	-0.58313311
177	Indoline	7.4368E-06	2.5656E-05	1.72572447	0.0016926	0.0142321	-1.7865365
287	Hypoxanthine	0.00078455	0.00116587	1.50034964	0.0419045	0.10866536	-0.57147111
323	DL-Stachydrine	0.00015326	0.00039038	1.55628925	0.0061699	0.03355176	-1.34888596
418	L(-)-Carnitine	0.0057199	0.00740708	1.61383771	0.00360026	0.02326267	-0.37291631

438	L-Methionine sulfoxide	0.00021999	0.00039543	1.77615331	0.00093441	0.01000301	-0.84597471
480	Gly-Val	2.5952E-05	0.00032063	2.08895405	0.00052107	0.0066279	-3.62697018
566	Methyl indole-3-acetate	2.1714E-05	3.48E-05	1.29669779	0.02386411	0.08040364	-0.68045264
633	Diphenylsulfoxide	0.00118257	0.00188706	1.43277919	0.01734411	0.06675471	-0.67421319
903	Hexanoylcarnitine	1.7046E-05	3.7131E-05	1.75273311	0.00182196	0.01485971	-1.12314886
919	N-Phenylacetylglutamine	8.0016E-05	0.0002279	1.06182482	0.04490378	0.11318521	-1.51003958
978	α-Aspartylphenylalanine	1.375E-05	8.8496E-05	1.86049828	0.0077634	0.03911087	-2.68619537
1006	Octanoyl-L-Carnitine	8.0769E-05	0.00019033	1.79799881	0.00102705	0.01061784	-1.23661784
1112	Decanoylcarnitine	0.00012416	0.00034241	1.82372623	0.00072123	0.00840764	-1.46347309
1192	Lauroylcarnitine	5.0077E-05	0.0001048	1.50834168	0.01049559	0.04859415	-1.06543568
210	raspberry ketone	2.2337E-05	4.8411E-05	1.80090448	0.00111181	0.01065569	-1.11591752
241	Gly-Val	1.2642E-05	0.00016381	2.04493356	0.00034208	0.00576618	-3.69572045
254	N-Formyl-DL-methionine	2.9003E-05	3.9107E-05	1.50049182	0.02546013	0.06360991	-0.4312306
349	2-Hydroxyhippuric acid	4.6116E-05	0.00019033	1.35664642	0.01579322	0.04827779	-2.04515558
364	(-)-Camphanic acid	3.393E-05	5.7041E-05	1.33862181	0.02582536	0.06414586	-0.74947
378	Lauric acid	1.9159E-05	2.2713E-05	1.2351636	0.03671149	0.07836572	-0.24554827
421	Kynurenine	3.6445E-05	4.6393E-05	1.29549251	0.03330667	0.0740962	-0.34816903
830	α-Aspartylphenylalanine	2.5247E-05	0.0001352	1.80001342	0.00954379	0.03520232	-2.42088845
960	Tretinoin	0.00023574	0.00036836	1.25481284	0.03997012	0.08214036	-0.64395648
2659	Levothyroxine	1.555E-05	1.8537E-05	1.24489172	0.03312075	0.07385266	-0.2535037

Supplementary Table 11 List of specific primers used in this study.

Bacteria	Primer	Function	Reference
P.acidilactici	F：5’-GGACTTGATAACGTACCCGC-3’<br>R：5’-GTTCCGTCTTGCATTTGACC-3’	Specific primer for GR-1	(Mora, Fortina et al. 1997)
Total bacteria	F: 5’-GCAGGCCATACACATGCAAGTC-3’<br>R: 5’-CTGCTGCCTCCCGTAGGAGT-3’	Universal primer for total bacteria	(Castillo, Martín-Orúe et al. 2006)
V3&V4 region	F：5’- CCTACGGGNGGCWGCAG-3’<br>R：5’ -GACTACHVGGGTATCTAATCC-3’	For 16S rRNA gene sequencing	(Klindworth, Pruesse et al. 2012)

SUPPLEMENTARY FIGURE

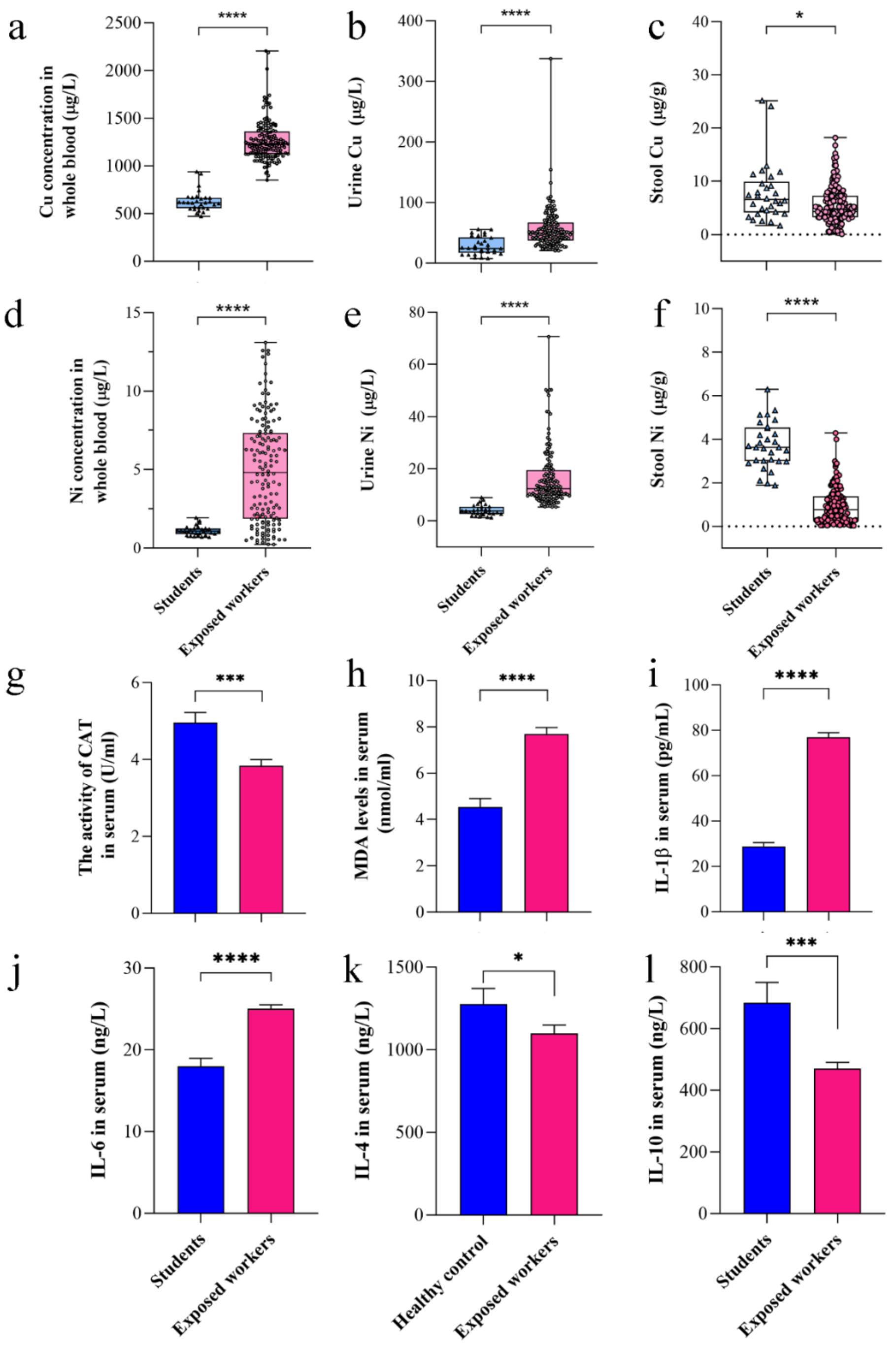

Supplementary Figure 1. Boxplots of Cu and Ni levels in blood, urine and faeces of all participants at baseline. The concentration of Cu/Ni in whole blood (**a, d**), urine (**b, e**), and faeces (**c, f**) of students and exposed workers. Students, n=30; Exposed

workers, n=152. (**g**) The activity of CAT, (**h**) MDA level, (**i**) IL-1β, (**j**) IL-6, (**k**) IL-4 and (**l**) IL-10 in serum of all participants at baseline. In the box plot, box limits represent 25 and 75 percentiles; line within box indicates median; the whisker ends the points minimum and maximum. Data are represented as mean± SEM. Statistical significance was done by two-tailed Mann-Whitney test (*$p < 0.05$, **$p < 0.01$, ***$p < 0.001$, ****$p < 0.0001$) using the GraphPad Prism (version 8.0.1). Source data are provided as a Source Data file.

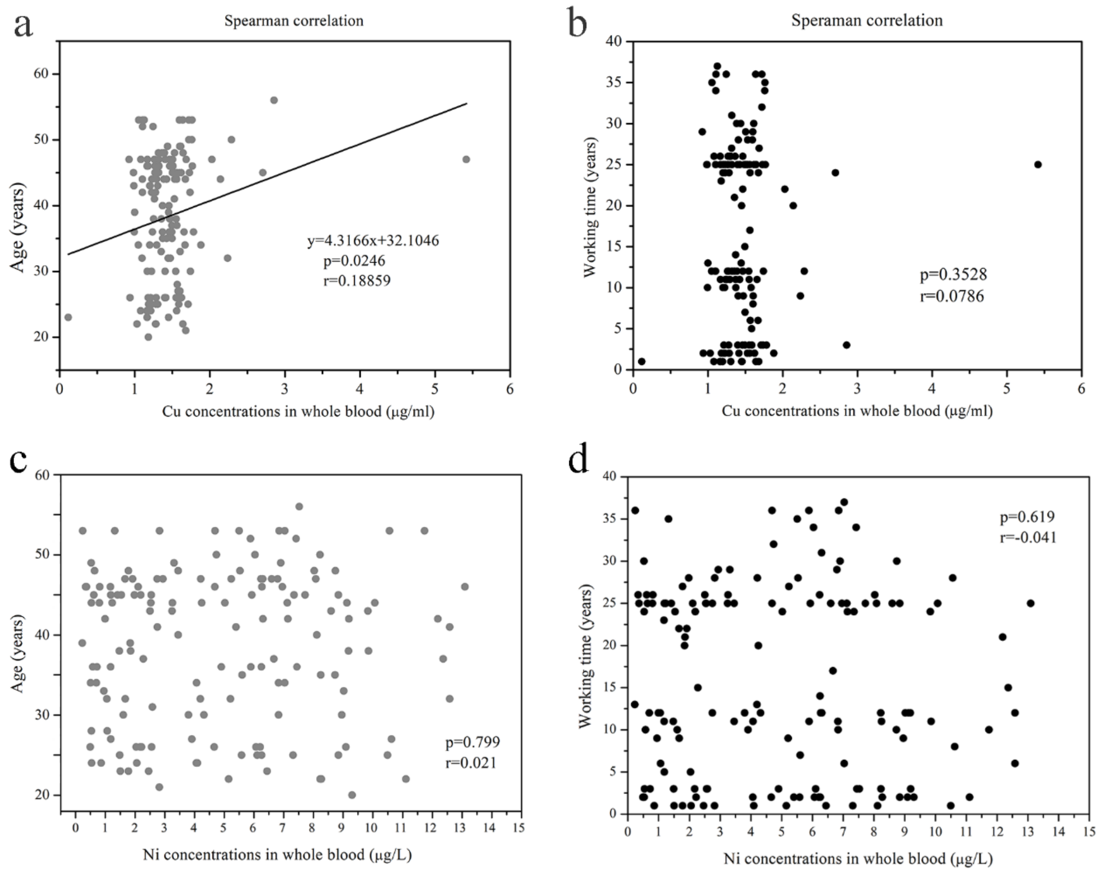

Supplementary Figure 2. Spearman's correlation of the Cu and Ni levels in blood with respect to age (**a, c**) and working time (**b, d**). Correlation analysis was conducted using SPSS 21 software and graphs were generated using Origin Pro 8.

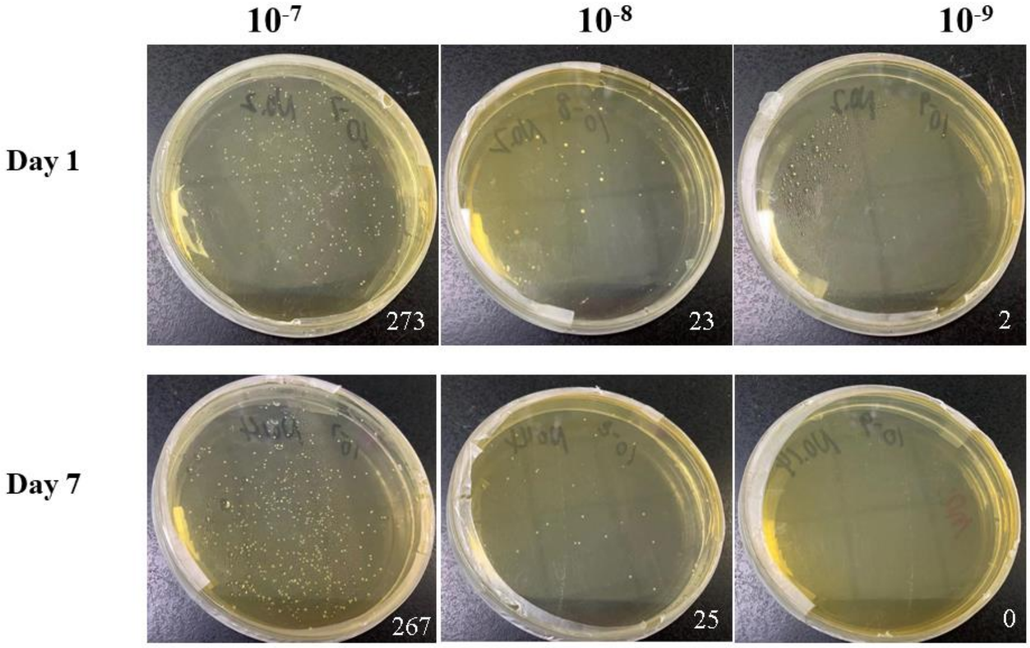

Supplementary Figure 3. Evaluation of viability of probiotic in yogurt within 7 days of storage at 4 ℃. The viability of probiotic in 1 gram of yogurt was evaluated by pouring plate method in MRS solid medium at 37 ℃ for 72h.

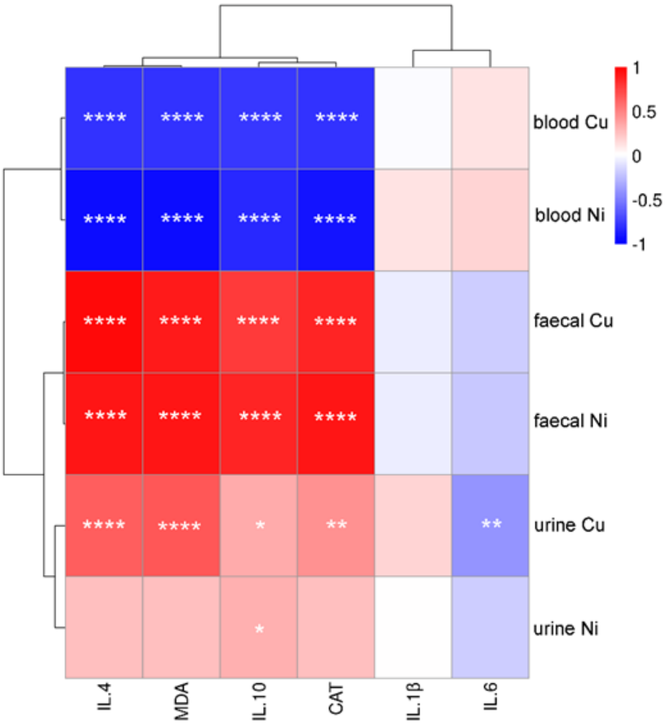

Supplementary Figure 4. Spearman's correlation analysis of the reduction of heavy metals load between the alteration of physiological parameters with probiotic yogurt administration. *p<0.05, **p<0.01, ****p<0.0001.

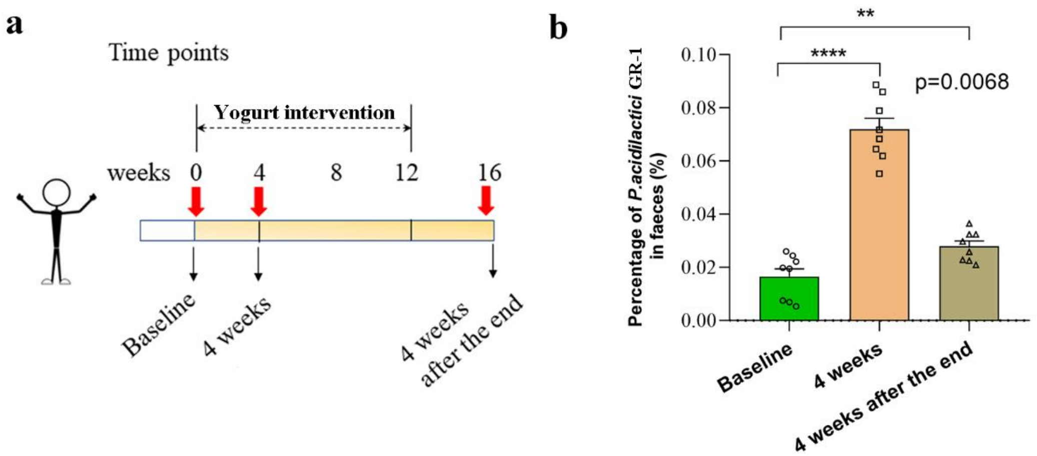

Supplementary Figure 5. Probiotic GR-1 colonization in the intestine and concentration of SCFAs in the faeces of occupational workers after 12 weeks of probiotic yogurt intervention. a Schematic illustration of the probiotic yogurt intervention to workers. Fecal samples were analyzed at baseline, 4 weeks after intervention, and 4 weeks after the end. **b** Changes in *Pediococcus acidilactici* load in feces throughout the experiment. The relative abundance of GR-1 is expressed as the ratio of the absolute copies of GR-1to total bacteria (probiotic yogurt (n=8) vs. baseline (n=8)). Data are represented as mean± SEM. Statistical analysis was done by one-way ANOVA with Tukey' s post-hoc analysis. Source data are provided as a Source Data file.

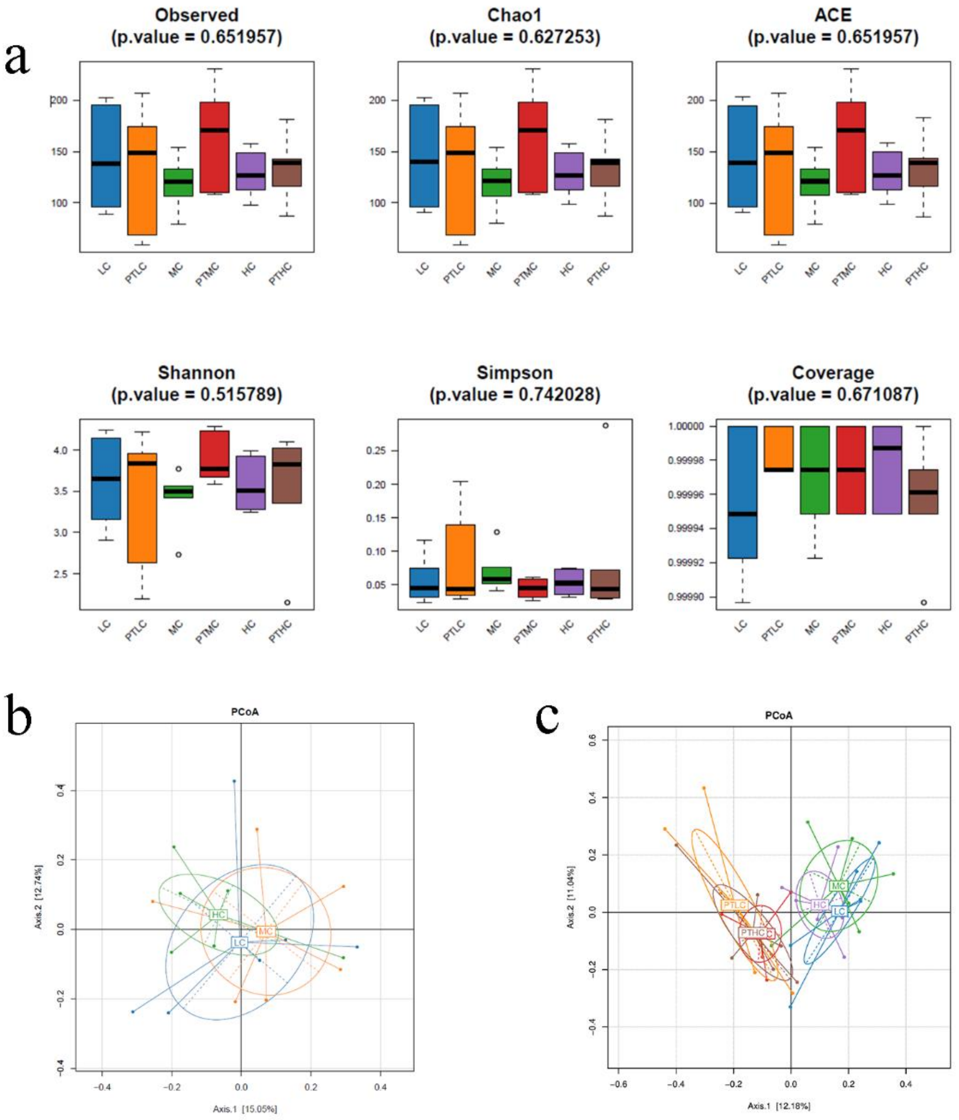

Supplementary Figure 6. Effect of probiotic yogurt on the gut microbiome of workers using 16S rRNA gene sequencing. (**a**) Alpha diversity (Observed, Chao1, ACE, Shannon, Simpson, and Coverage) of gut microbiota; Principal component ordination analysis (PCoA) of gut microbiota (**b** and **c**) among groups at baseline and at the end of the study (after 12 weeks of probiotic yogurt intervention) with low, medium and high levels of blood heavy metals. LC, low levels; MC, medium levels; HC, high levels; PTLC, low levels after 12 week of probiotic yogurt intervention; PTMC, medium levels after 12 week of probiotic yogurt intervention; and PTHC, high levels after 12 week of probiotic yogurt intervention. For a, box limits represent 25 and 75 percentiles; the line within the box indicates median; the whisker represents the minimum and maximum points. Alpha diversity estimates were calculated using Kruskal-Wallis test and PCoA plots were performed based on bray-Curtis distance of

ASVs.

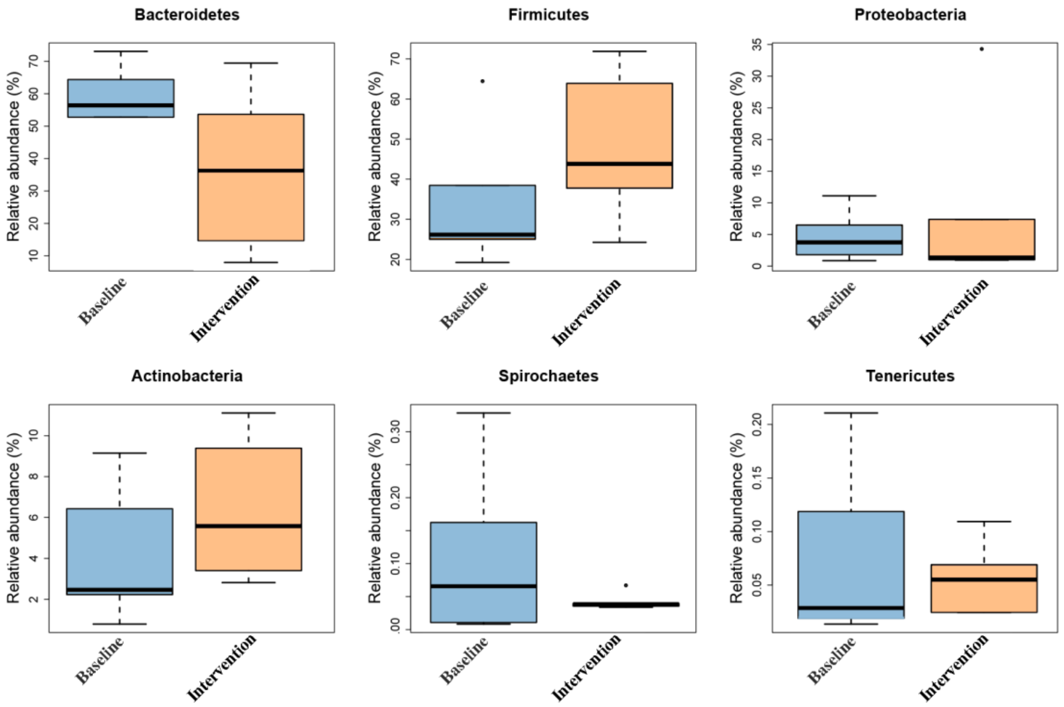

Supplementary Figure 7. Effect of probiotic yogurt on phylum level using metagenomic sequencing. Baseline and Intervention indicate gut microbiome of workers at the time of recruit and after 12 weeks of probiotic yogurt intervention, respectively. Box limits represent 25 and 75 percentiles; the line within the box indicates median; the whisker represents the minimum and maximum points. Statistical analysis of phylum was performed using Metastats test.

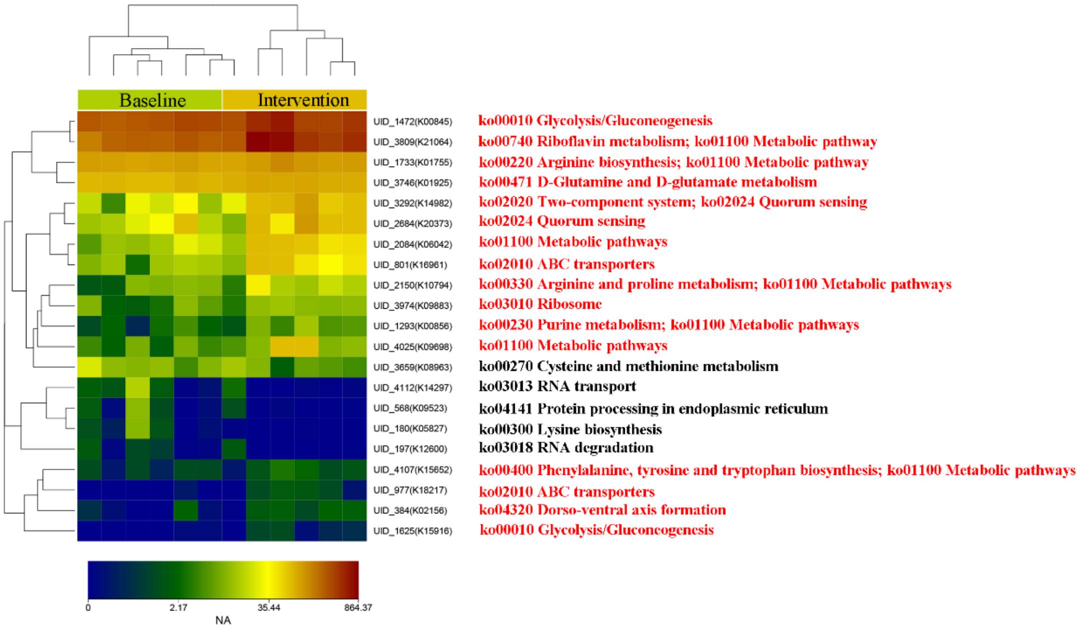

Supplementary Figure 8. Heatmap of KEGG pathway enrichment analysis for intervention versus baseline group. Red text indicates pathway enrichment in the intervention group, and black text represents pathway enrichment in the baseline group. KEGG pathway that significantly differed in abundance between the two groups were calculated using Wilcoxon rank-sum test after Benjamini-Hochberg adjustment. In the heatmap, red represents significant enrichment.

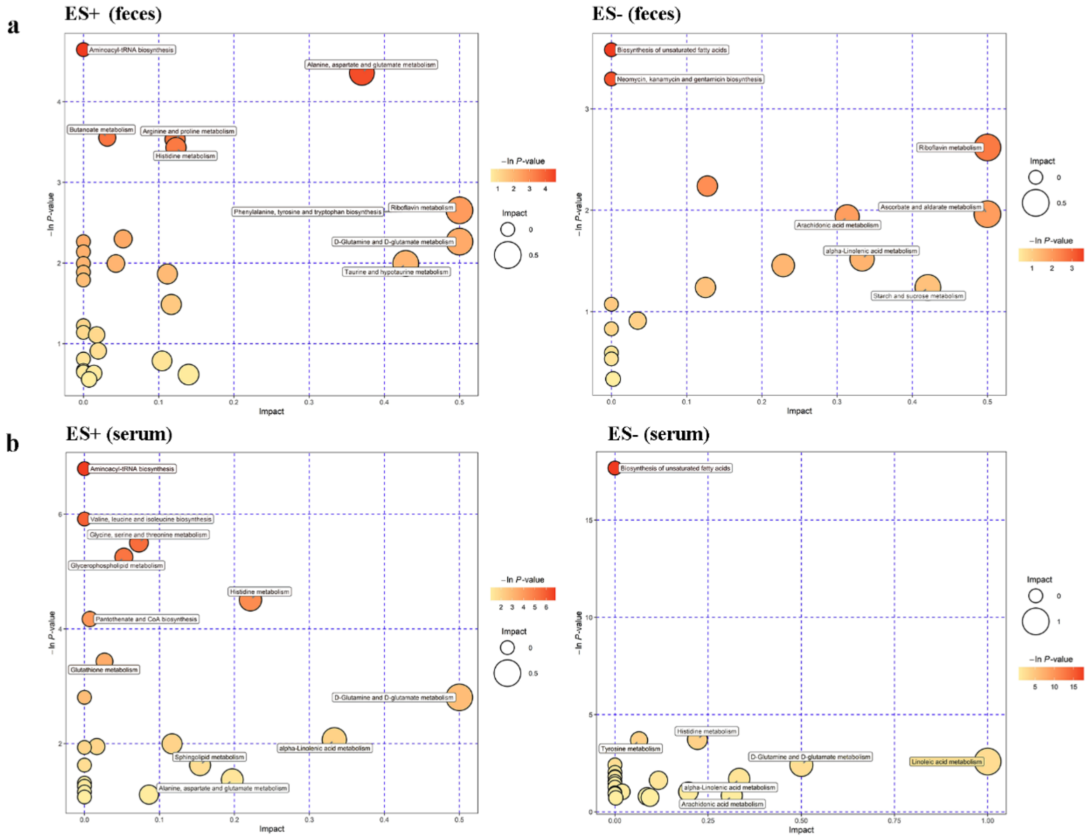

Supplementary Figure 9. Bubble plot of enriched pathways analysis in faeces (**a**) and serum samples (**b**) of workers with probiotic yogurt intervention vs baseline.

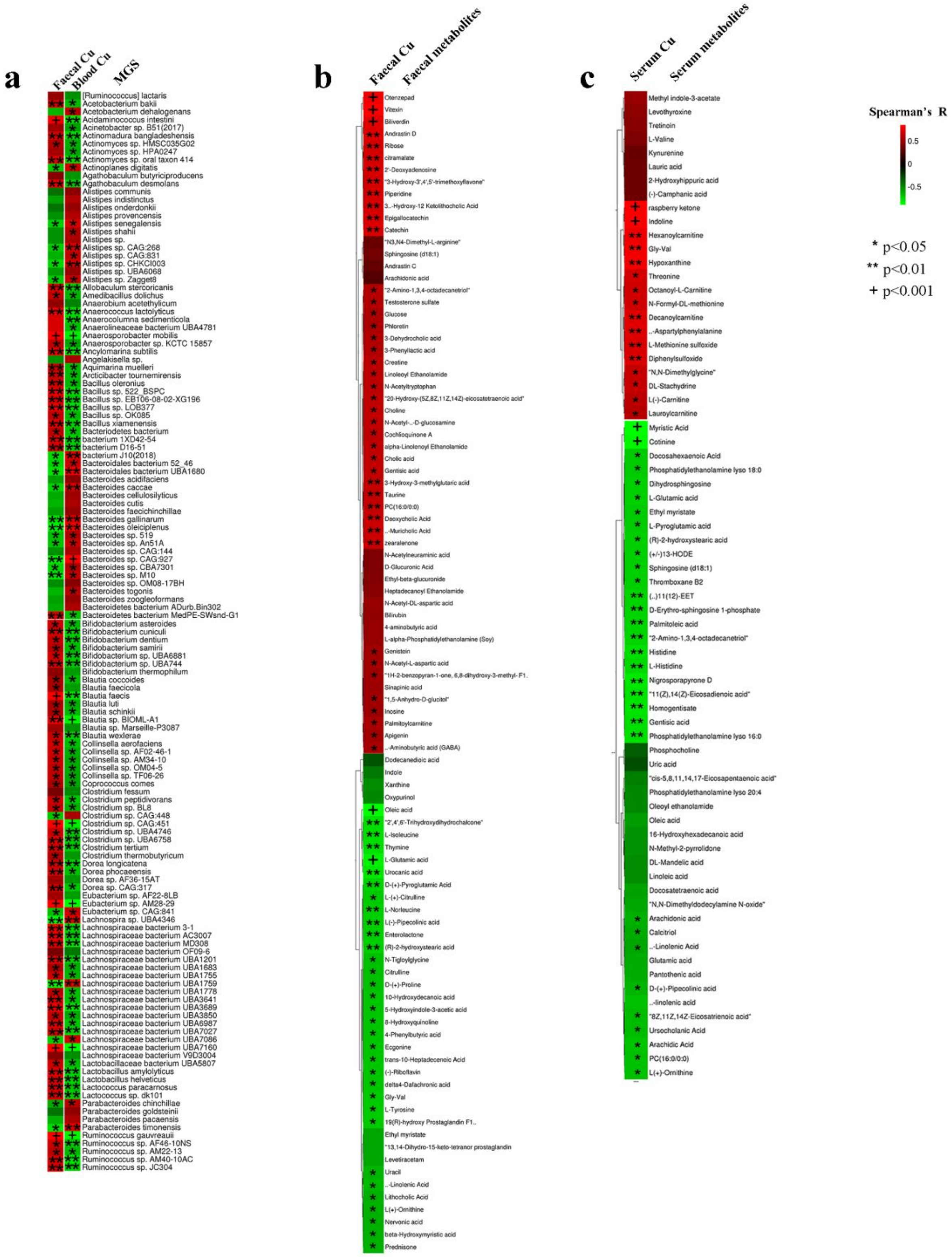

Supplementary Figure 10. Spearman's correlation of the members in differential MGSs, faecal metabolites or serum metabolites with blood Cu concentration or faecal Cu concentration. In the heatmap, the legend denotes correspondingly the R value of Spearman.

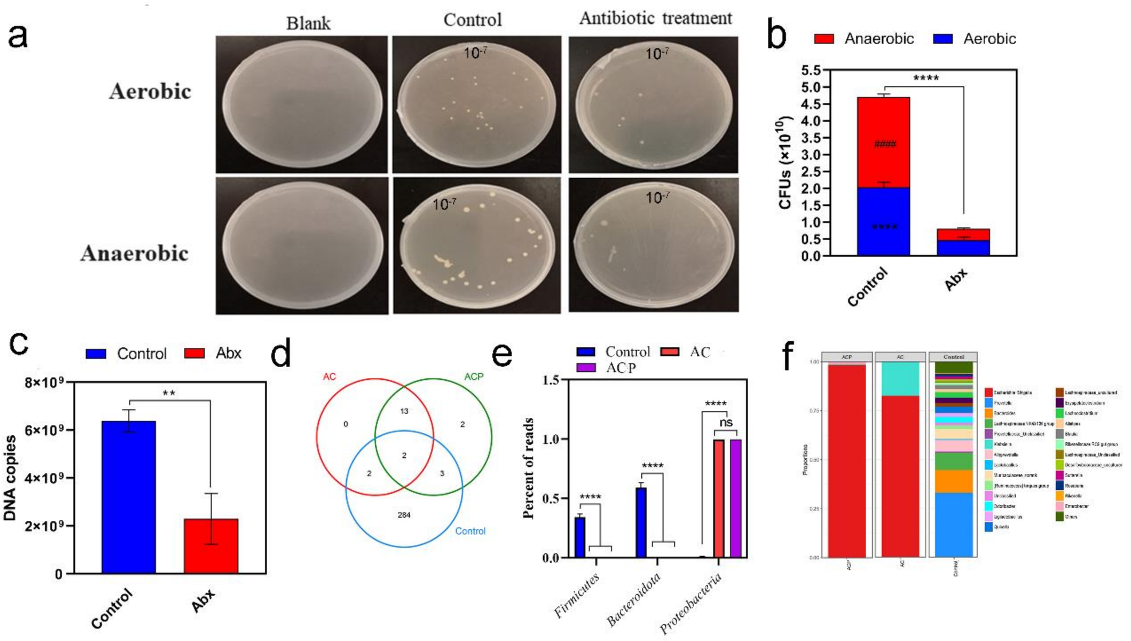

Supplementary Figure 11. The effects of antibiotic treatment on gut microbiota of mice after 10-days treatment. a The spread plate counting of culturable faecal microbes at a dilution ratio of 1: 10, 000, 000 under aerobic and anaerobic conditions. **b** Statistics of the number of viable bacteria in fresh faeces under aerobic and anaerobic conditions. **c** The absolute quantification of 16S rDNA copies in faeces of the control group and the antibiotics-treated groups**. d** Venn diagram showing the overlapping of OUT among the control group and two antibiotics-treated groups (AC, Abx+Cu; ACP, Abx+Cu+Pro) on day 10. **e)** The relative abundance of GM in mice at the phylum level. The percentage of Firmicutes and Bacteroidetes reads in the two groups treated with antibiotics (the percent of Proteobacteria in the control group was too low to display in figure). **f)** The relative abundance of GM in mice at the genus level. For b and c, statistical analysis was done by Wilcoxon rank-sum test. Data are represented as mean± SEM in figure b, c and e. For e, statistical analysis was done by an ordinary one-way ANOVA with Tukey' s post-hoc analysis. (*$p < 0.05$, **$p < 0.01$, ***$p < 0.001$, ****$p < 0.0001$, ns represents not significant).

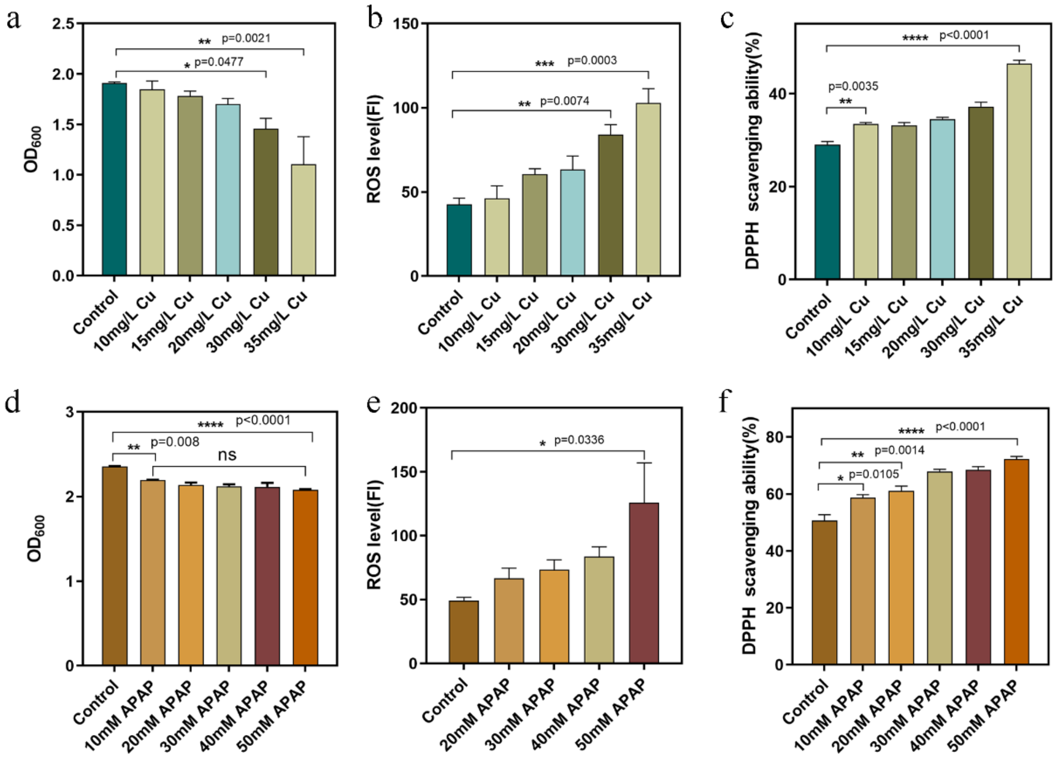

Supplementary Figure 12. The antioxidant ability of probiotic *Pediococcus acidilactici* GR-1 against Cu and APAP. The average optical density of GR-1 in medium with **(a)** Cu gradient concentration and **(d)** APAP gradient concentration after 12h of co-culture. ROS level of the supernatant after co-culture of **(b)** GR-1 and Cu and **(e)** GR-1 and APAP for 12h. DPPH scavenging ability of supernatant after co-culture of **(c)** GR-1 and Cu and **(f)** GR-1 and APAP for 12h. Data are represented as mean± SEM. Statistical analysis was done by one-way ANOVA with Tukey's post-hoc analysis (*$p < 0.05$, **$p < 0.01$, ***$p < 0.001$, ****$p < 0.0001$, ns represents not significant).

References

Castillo, M., S. M. Martín-Orúe, E. G. Manzanilla, I. Badiola, M. Martín and J. Gasa (2006). "Quantification of total bacteria, enterobacteria and lactobacilli populations in pig digesta by real-time PCR." Veterinary Microbiology **114**(1): 165-170.

Klindworth, A., E. Pruesse, T. Schweer, J. Peplies, C. Quast, M. Horn and F. O. Glöckner (2012). "Evaluation of general 16S ribosomal RNA gene PCR primers for classical and next-generation sequencing-based diversity studies." Nucleic Acids Research **41**(1): e1-e1.

Mora, D., M. G. Fortina, C. Parini and P. L. Manachini (1997). "Identification of Pediococcus acidilactici and Pediococcus pentosaceus based on 16S rRNA and ldhD gene-targeted multiplex PCR analysis." FEMS Microbiology Letters **151**(2): 231-236.

Saravanabhavan, G., K. Werry, M. Walker, D. Haines, M. Malowany and C. Khoury (2017). "Human biomonitoring reference values for metals and trace elements in blood and urine derived from the Canadian Health Measures Survey 2007–2013." International Journal of Hygiene and Environmental Health **220**(2, Part A): 189-200.
